# Comprehensive Characterization of Bile Acids in Human Biological Samples and Effect of 4-Week Strawberry Intake on Bile Acid Composition in Human Plasma

**DOI:** 10.3390/metabo11020099

**Published:** 2021-02-10

**Authors:** Anqi Zhao, Liyun Zhang, Xuhuiqun Zhang, Indika Edirisinghe, Britt M. Burton-Freeman, Amandeep K. Sandhu

**Affiliations:** Department of Food Science and Nutrition and Center for Nutrition Research, Institute for Food Safety and Health, Illinois Institute of Technology, Chicago, IL 60616, USA; azhao2@hawk.iit.edu (A.Z.); lzhan134@hawk.iit.edu (L.Z.); xzhan198@iit.edu (X.Z.); iedirisi@iit.edu (I.E.); bburton@iit.edu (B.M.B.-F.)

**Keywords:** bile acid, polyphenols, strawberry, human, plasma, urine, feces, microbial metabolites, UHPLC-Q-TOF, UHPLC-QQQ

## Abstract

Primary bile acids (BAs) and their gut microbial metabolites have a role in regulating human health. Comprehensive characterization of BAs species in human biological samples will aid in understanding the interaction between diet, gut microbiota, and bile acid metabolism. Therefore, we developed a qualitative method using ultra-high performance liquid chromatography (UHPLC) coupled with a quadrupole time-of-flight (Q-TOF) to identify BAs in human plasma, feces, and urine samples. A quantitative method was developed using UHPLC coupled with triple quadrupole (QQQ) and applied to a previous clinical trial conducted by our group to understand the bile acid metabolism in overweight/obese middle-aged adults (*n* = 34) after four weeks strawberry vs. control intervention. The qualitative study tentatively identified a total of 81 BAs in human biological samples. Several BA glucuronide-conjugates were characterized for the first time in human plasma and/or urine samples. The four-week strawberry intervention significantly reduced plasma concentrations of individual secondary BAs, deoxycholic acid, lithocholic acid and their glycine conjugates, as well as glycoursodeoxycholic acid compared to control (*p* < 0.05); total glucuronide-, total oxidized-, total dehydroxyl-, total secondary, and total plasma BAs were also lowered compared to control (*p* < 0.05). The reduced secondary BAs concentrations suggest that regular strawberry intake modulates the microbial metabolism of BAs.

## 1. Introduction

Bile acids (BAs) are well known for their essential role in lipid digestion and absorption, stimulating the flow of bile and promoting the secretion of cholesterol from the liver [1]. In the last 10 years, BAs have been recognized as having a role in metabolic processes, such as glucose, lipid, and energy metabolism, gut microbiota structure and function, along with hepatic and colonic inflammation through activation of the farnesoid X receptor (FXR) and G-protein coupled receptor 5 (TGR5) [2,3,4,5]. Primary BAs, including chenodeoxycholic acid (CDCA) and cholic acid (CA), are synthesized from cholesterol in the liver and stored in the gallbladder as glycine or taurine conjugates. Bile acids are released into the small intestinal lumen to facilitate the emulsification of lipids after consuming a meal. Following lipids absorption, around 95% of BAs are reabsorbed in the terminal ileum via enterohepatic circulation [6]. The remaining BAs undergo gut microbial biotransformation to form secondary BAs, mainly lithocholic acid (LCA) and deoxycholic acid (DCA) in the colon. Most of the secondary BAs are absorbed from the colon, although LCA is preferentially excreted via feces [7]. Changes in BAs metabolism, such as higher rate of conversion of primary to secondary BAs and elevated urinary BAs excretion, have been observed in people with type 2 diabetes, obesity, and Alzheimer diseases [8,9,10,11,12]. These observations may be a result of altered gut microbiota in these groups.

In recent years, research has focused on BAs transforming gut microbiota and how gut dysbiosis may alter the BA pool to influence local gut health and other diseases. Comprehensive profiling of BA species under different experimental conditions will help researchers to understand the complex interactions between gut microbiota, BA biotransformation, and host health. Several studies have characterized BA species in one or two types of human biological samples, such as plasma, urine, or feces, using liquid chromatography and mass spectrometry [13,14,15,16,17,18,19]. The major primary and secondary BAs species (i.e., CA, CDCA, DCA, LCA) and their taurine-, glycine- or sulfate-conjugates were identified. However, studies analyzing BAs in plasma, urine, and feces are scarce. Characterization of BAs in all these types of samples will provide a more comprehensive view of BA species and their metabolic fate, leading to a better understanding of BA metabolism influencing host health.

Berries rich in polyphenolic components possess anti-inflammatory and antioxidant effects and have been associated with improvements in metabolic syndrome, endothelial dysfunction, cardiovascular health, and diabetes [20]. Dietary polyphenols reaching the colon may benefit host health by modulating microbiota composition and metabolism, and impacting immunity [21,22]. The changes brought by dietary polyphenols on gut microbiota may in turn influence the microbial transformation of BAs, thereby modulating the BA pool. However, only a few studies have investigated the influence of dietary polyphenols on BA metabolism [23,24,25,26].

The primary aim of our study was to comprehensively characterize BA species in human biological samples, including plasma, feces, and urine. Further, we developed a method to quantify BAs and their metabolites in plasma samples after four-week strawberry consumption.

## 2. Results

### 2.1. Identification of BA Species in Human Biological Samples Using UHPLC-Q-TOF

**Figure 1:**
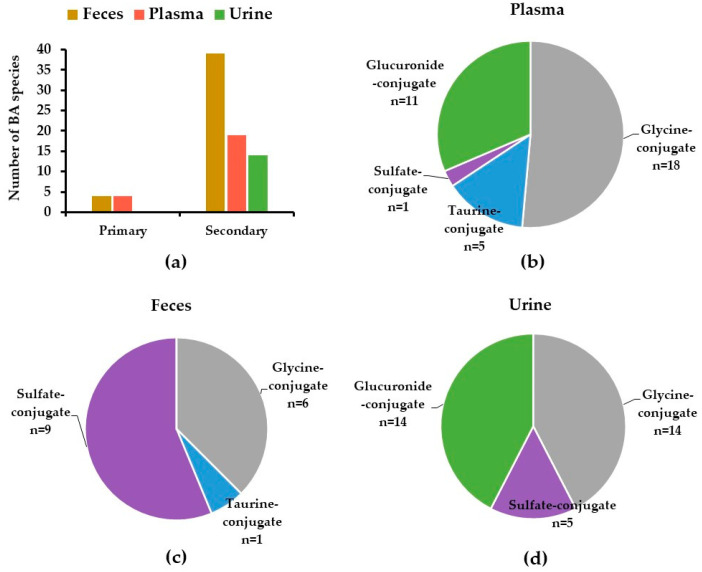
Distribution of BAs in human feces, plasma, and urine samples: (**a**) primary and secondary BAs; (**b**–**d**) different BAs subgroups, categorized based on their conjugates. Data are presented as number of BA species detected in each subgroup of BAs.

Out of 81 tentatively identified BAs and their metabolites in the human biological samples, 37, 49, and 31 BA species were characterized in plasma, feces, and urine samples, respectively (Table 1). Plasma samples contained a diverse array of BA species, including primary, secondary BAs, and their glycine-, taurine-, sulfate-, and glucuronide-conjugates, whereas fecal samples mostly contained secondary BAs and sulfate-conjugates. BA conjugates, including glucuronide-, glycine-, taurine-, and sulfate-conjugates were characterized in urine samples (Table 1, Figure 1).

### 2.2. Optimization of Chromatographic Conditions for BAs Separation and Quantification Using UHPLC-QQQ

**Figure 2:**
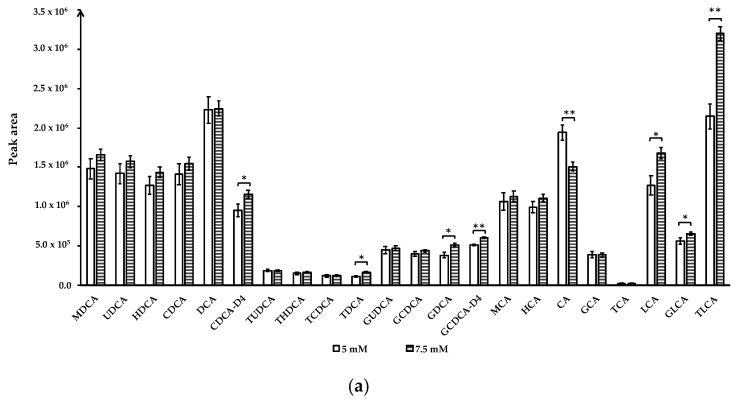

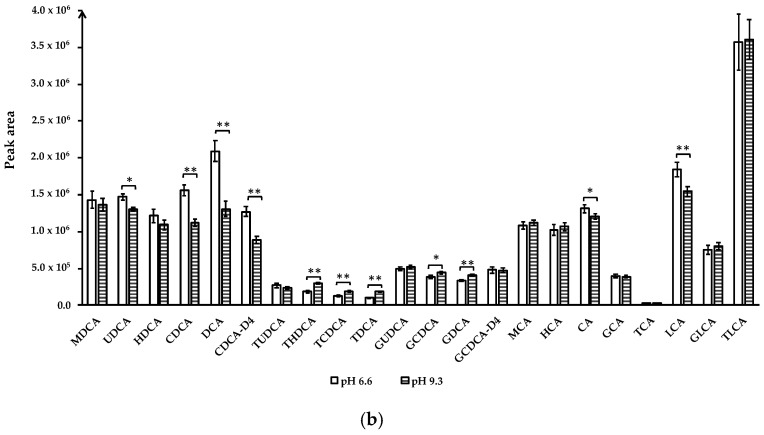
Effect of ammonium acetate concentration and mobile phase A (0.01% formic acid in water) pH on bile acids peak area. (**a**) Ammonium acetate concentration; (**b**) mobile phase A pH. Data for ammonium acetate concentration at 1 mM, 10 mM and mobile phase A pH 3.8 not shown due to co-elution of TUDCA and THDCA. Data are presented as mean and standard deviation based on three measurements. * *p* < 0.05, ** *p* < 0.01. Abbreviations: murideoxycholic acid (MuriDCA), ursodeoxycholic acid (UDCA), hyodeoxycholic acid (HDCA), chenodeoxycholic acid (CDCA), deoxycholic acid (DCA), chenodeoxycholic acid-D4 (CDCA-D4), tauroursodeoxycholic acid (TUDCA), taurohyodeoxycholic acid (THDCA), taurochenodeoxycholic acid (TCDCA), taurodeoxycholic acid (TDCA), glycoursodeoxycholic acid (GUDCA), glycochenodeoxycholic acid (GCDCA), glycodeoxycholic acid (GDCA), glycochenodeoxycholic acid-2,2,4,4-D4 (GCDCA-D4), β-muricholic acid (β-MCA), hyocholic acid (HCA), cholic acid (CA), glycocholic acid (GCA), taurocholic acid (TCA), lithocholic acid (LCA), glycolithocholic acid (GLCA), taurolithochoic acid (TLCA).

Ammonium acetate concentrations: To optimize the quantitative analysis method, we compared the effect of ammonium acetate at 1 mM, 5 mM, 7.5 mM, and 10 mM in mobile phase A on BAs separation and detection. Mobile phase A with 1 mM and 10 mM ammonium acetate were unable to separate TUDCA and THDCA peaks, even though larger peak areas were obtained for several BA compounds when eluted by ammonium acetate buffer at these two concentrations compared to 5 mM or 7.5 mM (data not shown). BAs were well-separated with 5 mM and 7.5 mM ammonium acetate in mobile phase A and 7.5 mM ammonium acetate buffer yielded larger peak areas for CDCA-D4, TDCA, GDCA, GCDCA-D4, LCA, GLCA, and TLCA (*p* < 0.05). Only the peak area of CA increased at ammonium acetate concentration of 5 mM (*p* < 0.01) (Figure 2a).

Altering mobile phase pH: To understand the effect of mobile phase pH on BAs separation and detection, we compared mobile phase A containing 7.5 mM ammonium acetate by adjusting pH to 3.8, 6.6, and 9.3 using acetic acid (to pH = 3.8) or ammonium hydroxide (to pH = 9.3). Mobile phase A with pH 3.8 did not separate the TUDCA and THDCA peaks (data not shown), whereas pH 6.6 and 9.3 obtained good resolution for all analytes. Similar results have been reported by other studies [15,16]. Comparison of pH 6.6 and 9.3 showed that the peak areas of unconjugated bile acids, including UDCA, CDCA, DCA, CDCA-D4, CA, and LCA, were significantly increased at pH = 6.6, while THDCA, TCDCA, TDCA, GCDCA, GDCA had higher peak areas at pH = 9.3 (*p* < 0.05) (Figure 2b). Therefore, mobile phase A containing 7.5 mM ammonium acetate with pH adjusted to 6.6 was chosen for quantitative analysis of bile acids.

### 2.3. Method Validation

The analytical method for the quantitation of BAs in human plasma using UHPLC-QQQ was validated in terms of linearity, sensitivity, recovery, matrix effect, and precision (Table 2).

The calibration curves were prepared in the solvent and blank plasma with seven series dilution levels of the mixture of BA standard solution. All BA standards had good linearity with correlation coefficient (R^2^) > 0.99 (Table 2).

Most BAs were in the LOQ ranging from 2.5–6.6 nmol/L and LOD ranging from 0.2–3.3 nmol/L. Compared to other compounds, cholic acid was at a much higher LOD (6.1 nmol/L) and LOQ (49.0 nmol/L), which may be due to ionization suppression caused by ammonium acetate in the mobile phase.

The recoveries were calculated as the ratio of the measured area of the mixture of BA standards at low (31.25 ng/mL) and high (1000 ng/mL) concentrations in blank human plasma, prepared before and after the SPE procedures. The recovery evaluated the impacts of the sample preparation and extraction procedures on the concentrations of BAs. The recoveries of all BA standards ranged between 77–151% at high concentration and 74–107% at low concentration (Table 2).

As for matrix effect, out of 20 BA standards, 8 compounds showed negative matrix effect. Eleven BAs showed absolute matrix effects within 10%, eight of BAs obtained absolute matrix effects between 10–25%, and only one compound (GCA) had an absolute matrix effect above 30% (Table 2). Two internal standards, CDCA-D4 and GCDCA-D4, were used to minimize the matrix effects.

The intra- and inter-day precisions were in the range of 0.3–5.2% and 0.9–14.7%, respectively. Both inter- and intra-day precisions were within the acceptable criterion of <15%.

### 2.4. Application of the UHPLC-QQQ Methodology to a Human Study—Influence of Chronic Strawberry Supplementation on Bile Acid Metabolism

**Figure 3:**
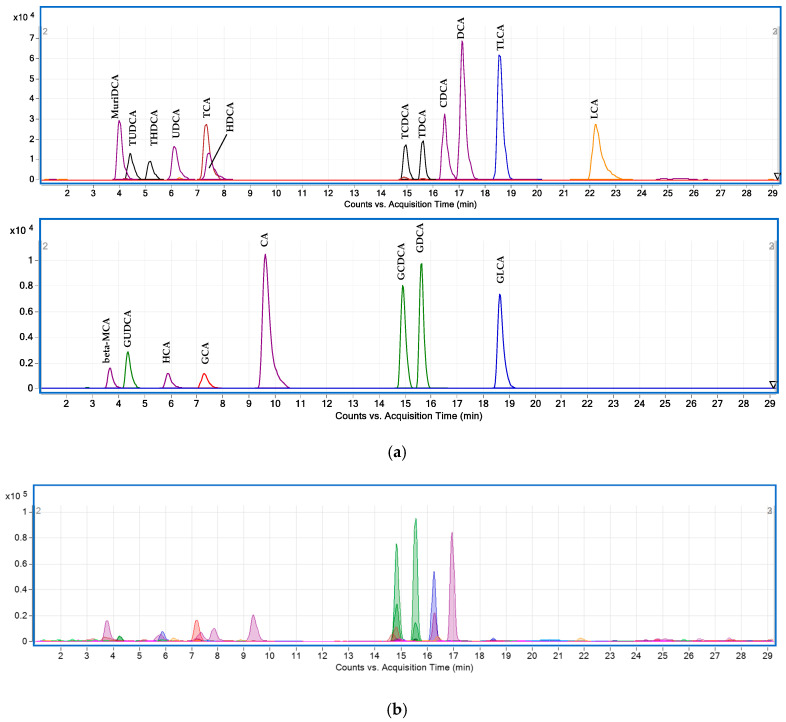
MRM chromatograms using UHPLC-QQQ: (**a**) BA standards in blank plasma; (**b**) BA compounds in human plasma. Abbreviations: cholic acid (CA), chenodeoxycholic acid (CDCA), deoxycholic acid (DCA), glycocholic acid (GCA), glycochenodeoxycholic acid (GCDCA), glycodeoxycholic acid (GDCA), glycolithocholic acid (GLCA), glycoursodeoxycholic acid (GUDCA), hyocholic acid (HCA), hyodeoxycholic acid (HDCA), lithocholic acid (LCA), β-muricholic acid (β-MCA), murideoxycholic acid (MuriDCA), taurocholic acid (TCA), taurochenodeoxycholic acid (TCDCA), taurodeoxycholic acid (TDCA), taurolithochoic acid (TLCA), tauroursodeoxycholic acid (TUDCA), taurohyodeoxycholic acid (THDCA), ursodeoxycholic acid (UDCA).

The validated UHPLC-QQQ method was applied to a four-week randomized, double-blind, placebo-controlled, crossover trial. A total of 100 BAs and their metabolites were tentatively identified in the plasma samples from 34 subjects. This larger number of BAs species identified by the quantitative method, compared to 81 BAs identified using UHPLC-Q-TOF, was due to more isomers detected in these samples (Table 3; Figure 3).

**Figure 4:**
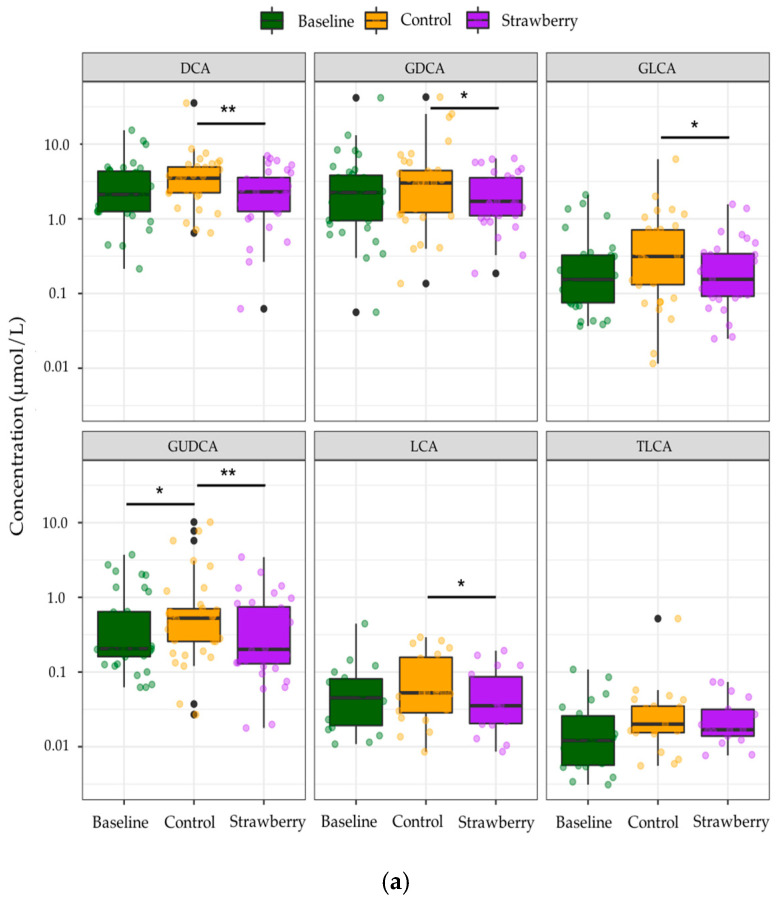

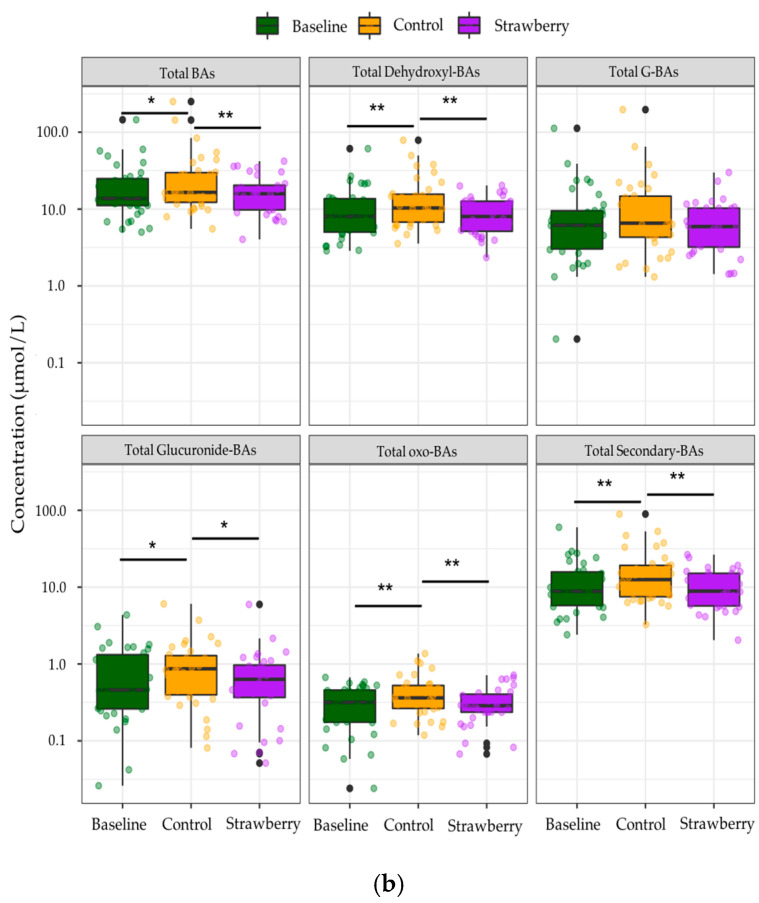
Changes in concentrations of (**a**) individual bile acids (**b**) bile acids in different subgroups after 4-week control or strawberry beverage intake compared to baseline (week 0). Data are presented as the mean and standard error of the mean. * *p* < 0.05, ** *p* < 0.01. The black dots are the outliers, which are defined as outside 1.5 times the interquartile range (IQR) above the upper quartile (Q3) and below the lower quartile (Q1) (Q1 − 1.5 × IQR or Q3 + 1.5 × IQR).

Among the identified compounds, only 85 BA species were quantified due to concentrations of the remainder of 15 compounds being below LOQ. Individual secondary BAs such as DCA, LCA, and their glycine conjugates as well as GUDCA were significantly decreased after four-week strawberry consumption compared to control (*p* < 0.05) (Figure 4a). BAs were categorized into different subgroups based on their conjugate groups, such as taurine and glycine, as well as the microbial transformation they have undergone, like dehydroxylation and oxidization by gut microbiota with 7α-dehydroxylase and hydroxysteroid dehydrogenases (HSDH) activities, respectively. A significant increase in concentrations of several subgroups of BAs, including total glucuronide-BAs, total oxo-BAs, total dehydroxyl-BAs, and total secondary BAs were observed after four-week control beverage intake compared to baseline/week 0 (*p* < 0.05). However, strawberry beverage intake (four weeks) retained the baseline concentrations of these subgroups of BAs (Strawberry vs Baseline, *p* > 0.05). The four-week strawberry intervention significantly reduced total glucuronide-, total oxo-, total dehydroxyl-, and total secondary BAs compared to control (*p* < 0.05) (Figure 4b). The total plasma BAs were also significantly reduced in the strawberry group compared to control (Figure 4b).

## 3. Discussion

In the present study, we tentatively identified 81 BA species in human biological samples using UHPLC-Q-TOF and investigated the influence of a four-week strawberry intervention on BA metabolism using UHPLC-QQQ. We, for the first time, characterized several glyco-BA glucuronide-conjugates and 3, 7, or 12- oxidized BA glucuronide-conjugates in human plasma and/or urine samples. We also observed that the four-week strawberry intervention significantly reduced plasma concentrations of several individual secondary BAs, such as DCA and LCA, as well as some BA subgroups, such as total secondary BAs (*p* < 0.05). Changes in these BA metabolites and subgroups suggest that regular strawberry intake modulates microbial metabolism of BAs.

**Figure 5:**
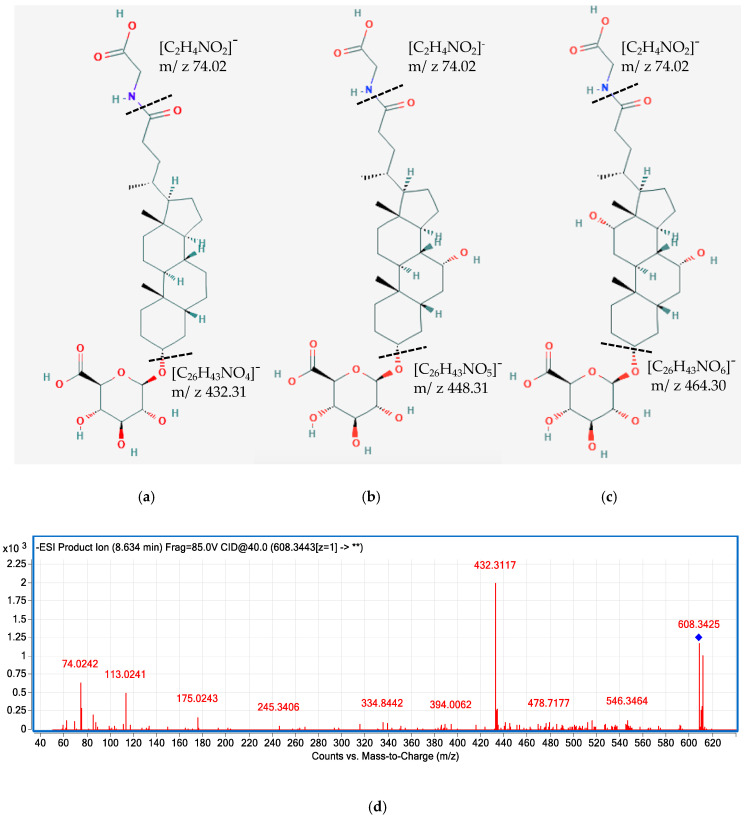

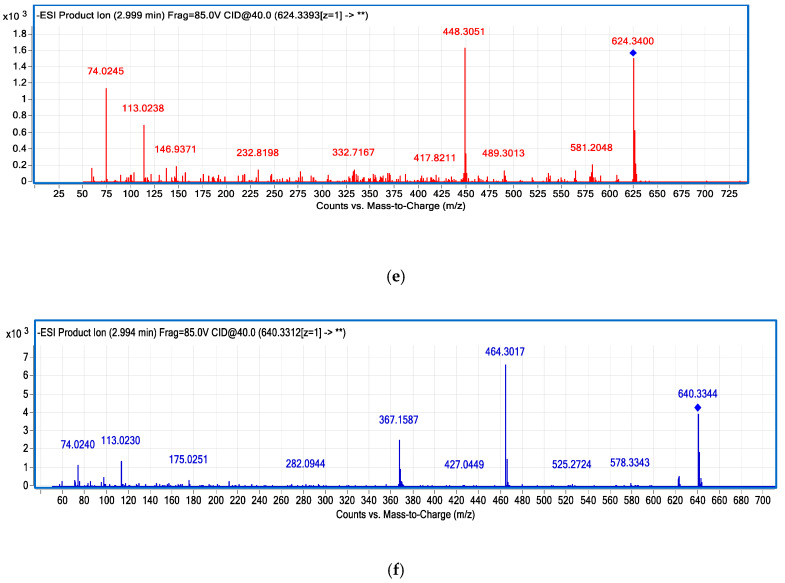
Tentatively proposed structure of glucuronide-GLCA (**a**), glucuronide-GCDCA (**b**), and glucuronide-GCA (**c**). MS/MS spectra of glucuronide-GLCA (*m*/*z* 608.34) (**d**), glucuronide-GCDCA (*m*/*z* 624.34) (**e**), and glucuronide-GCA (*m*/*z* 640.33) (**f**). The MS/MS spectra showing product ions 432.31, 448.31, 464.30 are corresponding to GLCA, GCDCA, and GCA moiety after the loss of the glucuronide group. The fragment 74.02 is corresponding to the conjugate base of glycine.

Most of the previous studies on the characterization of BAs in human samples only reported the major primary and secondary compounds, such as CDCA, DCA, CA, LCA, and their glycine-, taurine-, and sulfate-conjugates [15,30,31,32]. To the best of our knowledge, this is the first study to characterize a broad range of BA glucuronide conjugates in different human biological samples together with other common bile acids species. Besides identifying major BA glucuronide conjugates (i.e., glucuronide-CDCA, CA, and DCA), we characterized for the first time glucuronide-GLCA, GCDCA, and GCA in human plasma and/or urine samples using UHPC-Q-TOF, together with isomers of 3, 7, or 12- oxidized BA glucuronide-conjugates (Figure 5, Table 1). BAs are synthesized and secreted from the liver in the primary form, followed by microbial dehydrogenase transformation to generate the oxidized form, then they are reabsorbed from the gut and circulated back to the liver, where they could be glucuronidated by UDP-glucuronosyltransferases (UGTs) [33]. The glucuronide conjugates of secondary BAs could serve as a reflection of some gut microbiota enzymatic activities, which might be underestimated without characterizing these conjugates. Similarly, sulfate conjugates of 3, 7, or 12-oxidized and other secondary BAs were also tentatively identified in the present study, representing another portion of microbial metabolized BAs reflecting microbiota activities. Comprehensive characterization of secondary BAs and their conjugates would enable researchers to better understand the microbial metabolism of BAs.

**Figure 6:**
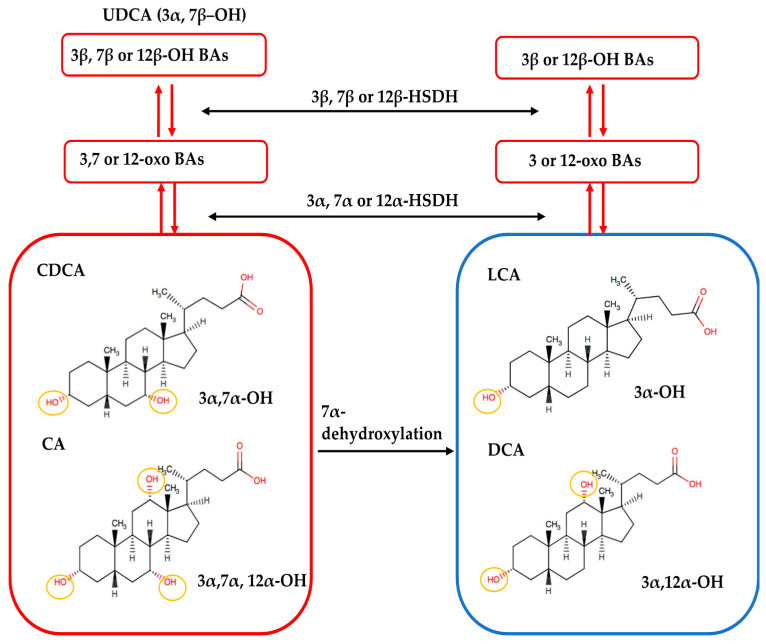
Microbial transformation of bile acids. CDCA and CA with 3, 7 and 3, 7, 12 α-hydroxyl groups are dehydroxylated by gut bacteria with 7α-dehydroxylase to lose the hydroxyl group at the 7-α carbon position, forming LCA and DCA. Gut bacteria can further oxidize the hydroxyl groups at 3, 7, and 12 α-carbon positions with hydroxysteroid dehydrogenases (HSDH) activity to form oxo-bile acids. The resulting oxo groups may then undergo epimerization and reduction to form 3, 7, or 12 β-hydroxyl groups. Figure modified based on Ikegami and Honda, 2018 [35].

Several secondary BAs were observed to have more than one isomer, which could be due to the diversity of biotransformation reactions performed by gut microbiota (Table 1). Gut microbes with 7α-dehydroxylase are capable of removing the 7α-hydroxyl group from CDCA and CA to form LCA and DCA, respectively (Figure 6). Hydroxysteroid dehydrogenases (HSDHs), another group of bacterial enzymes, catalyze the reversible oxidation of 3, 7, or 12 α-hydroxyl groups to form oxo-bile acids. The resulting oxo groups may further undergo epimerization and reduction to form 3, 7, or 12 β-hydroxyl groups [6,34]. Since these microbial enzymatic reactions result in hydroxyl- and oxo-groups at unfixed positions, it allows for the discovery of more than one novel isomers. For example, oxidation of CDCA can form 3-oxo CDCA or 7-OXO LCA; similarly, oxidation of CA can form 12-oxo CDCA, 7-oxo DCA or 3-oxo CA.

To maximize the detection of more BA species, we adapted the chromatographic conditions from Yin et al. for qualitative analysis of human biological samples. They reported that mobile phases with low formic acid concentrations (0.01% formic acid) worked better for all bile acid species analyzed in their study compared to mobile phases with formic acids at higher concentration or mobile phases containing ammonium acetate [36]. However, while analyzing BA standards mix solutions, we observed that the 0.01% formic acid mobile phase was not able to separate TUDCA and THDCA peaks, which may be due to high acidity of the mobile phase (pH = 3.08) as discussed below. To obtain optimal resolution for all BA peaks, we did not use the same chromatographic setting for quantitative analysis of human plasma samples.

The optimized mobile phases used for quantitative analysis of BAs by UHPLC-QQQ were: mobile phase A containing 7.5 mM ammonium acetate with pH adjusted to 6.6, and mobile phase B as 20% acetonitrile in methanol. The result for optimum concentration of ammonium acetate (7.5 mM) is consistent with the concentration used by Bathena et al. [15]. The addition of ammonium acetate to water/methanol (3:7, *v*/*v*) was found to increase the peak intensity (1.5-to 5-fold higher) for unconjugated and glycine/taurine-conjugated BAs than without addition of ammonium acetate to mobile phase [37]. The authors proposed that ammonium ions, as positively charged ions, may accelerate the dissociation of BA analytes into an anion to serve as counter ions for the ammonium within the charged fine droplets, which improved BAs ionization efficiency. In contrast, another study reported that the ionization of unconjugated BAs was inhibited by ammonium salts compared to formic acid as a buffer in the mobile phase. However, the effects of ammonium acetate at different concentrations on ionization were not investigated [36]. The result for the optimum pH of mobile phase A (pH = 6.6) is consistent with Alnouti et al., who also reported unconjugated bile acid-CDCA with higher signal intensity at pH 6 than pH 9 [19]. Unconjugated BAs have a pKa of approximately 6, whereas glycine- and taurine-conjugates have pKa around 4.5 and 1.5, which results in complete ionization of BAs at physiological pH and leads to increased solubility [38].

The quantitative method of analyzing BAs in human plasma using UHPLC-QQQ was validated as shown in Table 2. Most of the BA standards showed recoveries between 84–117% at high concentration and 82–107% at low concentration, which indicated the UHPLC-QQQ method offered accurate quantification for most of the BAs species. TCDCA, TCA, CA, and DCA showed recoveries >120% at high concentration, whereas THDCA, HDCA showed recoveries <80% at low concentration, which suggested enhancement/suppression of signal intensities in LC-MS/MS with electrospray ionization (ESI). Similar results showing BAs recoveries >120% or <80% have been reported by other researchers [13,39]. Matrix effect is known as the main interference for the reliability of quantitative results in LC-MS/MS with ESI method, due to the difficulty of endogenous compounds removal in human plasma [40,41]. The enhancement or suppression of ionization in ESI was evaluated by calculating the matrix effect. Two isotope-labeled internal standards were used to account for the matrix effects.

Reduced concentrations of secondary BAs such as DCA, LCA, and their glycine conjugates were observed after four weeks of strawberry intake compared to control (Figure 4a). Similar results have been reported in several in vivo and in vitro studies investigating the effects of polyphenol-rich diet on fecal BA excretion [23,24,25,26]. Increased DCA and LCA concentrations have been largely reported to be related to increased risk of colon cancer, probably through inducing the expression of proinflammatory factors, enhanced cell proliferation, and inducing the release of reactive oxygen species, which leads to DNA damage [42]. Reduced concentrations of DCA and LCA after four weeks of strawberry intervention observed in the present study suggest regular strawberry intake may modulate microbial metabolism of BAs, which could lead to a number of health benefits warranting further investigation, such as ameliorating colonic inflammation and reducing risk of colon cancer. In addition, compared to baseline, the control treatment significantly increased concentrations of several subgroups of bile acids, such as total oxo-BAs, total dehydroxyl-BAs, total secondary bile acids, and total plasma bile acids, whereas four-week strawberry consumption retained the baseline concentrations of these BAs subgroups. This could be related to metabolic health status of the individuals (overweight/obese) recruited for the study. Among this population (overweight/obese), total plasma BAs have been found to be positively correlated with body mass index (BMI) and negatively associated with cognitive restraint of eating [43]. Moreover, serum triglycerides levels in hyperlipidemic patients were linked to increased BAs synthesis rate [44]. It is possible that metabolically unhealthy individuals (overweight/obese) have a higher BA synthesis rate with increased concentrations of total BAs and these augmentations in BAs formation were prevented by the four-week strawberry intake in the present study. On the other hand, emerging evidence suggests that gut microbiota could be greatly altered under the conditions of obesity [45,46] and, more interestingly, reduction in the formation of secondary BAs was observed in germ-free mice after colonization of healthy human microbiota [47]. The four-week strawberry intervention might have modulated the gut microbiota community in these overweight/obese individuals, regulating the concentrations of secondary BAs subgroups unchanged compared to baseline, instead of being increased after four-week control beverage intake. Further analysis of compositional and functional changes in gut microbial community and biomarkers related to the host gut and metabolic health will provide more scientific evidence to bridge the knowledge gap between dietary polyphenols, bile acid metabolism, and the host health.

In conclusion, the present study developed a qualitative analysis method using UHPLC-Q-TOF and a quantitative method using UHPLC-QQQ to characterize a broad range of BA species in human biological samples. We observed significant reductions in the concentrations of several individual secondary BAs as well as different subgroups of BAs, such as total glucuronide-BAs and total dehydroxyl-BAs, after four-week regular strawberry intake compared to control. These data suggest strawberry consumption alters human bile acid metabolism, especially secondary bile acids related to colonic inflammation. The outcomes of this research warrants further investigation of different types of berries and dietary polyphenols on BAs metabolism and the subsequent influence on the host metabolic and gut health.

## 4. Materials and Methods

### 4.1. Chemicals and Reagents

Chenodeoxycholic acid (CDCA), ursodeoxycholic acid (UDCA), and hyodeoxycholic acid (HDCA) were purchased from Alfa Aesar by Thermo Fisher Scientific (Tewksbury, MA, USA). Glycodeoxycholic acid sodium salt (GDCA), glycochenodeoxycholic acid (GCDCA), taurodeoxycholic acid sodium salt (TDCA), taurochenodeoxycholic acid sodium salt (TCDCA), tauroursodeoxycholic acid (TUDCA), and taurocholic acid sodium salt hydrate (TCA) were purchased from Frontier Scientific (Logan, UT, USA). Murideoxycholic acid (MuriDCA), β-muricholic acid (β-MCA), glycoursodeoxycholic acid (GUDCA), taurolithochoic acid (TLCA), hyocholic acid (HCA) were purchased from Cayman Chemical (Ann Arbor, MI, USA). Lithocholic acid (LCA) was purchased from ApexBio Technology (Houston, TX, USA). Glycolithocholic acid (GLCA), glycochenodeoxycholic acid-2,2,4,4-D4 (GCDCA-D4), and chenodeoxycholic acid-D4 (CDCA-D4) were purchased from IsoSciences (Ambler, PA, USA). Deoxycholic acid (DCA) and cholic acid sodium salt (CA) were purchased from MP Biomedicals (Solon, OH, USA). Taurohyodeoxycholic acid sodium hydrate (THDCA) and glycocholic acid sodium salt (GCA) were purchased from Sigma-Aldrich (Saint Louis, MO, USA). Standard stock solutions were prepared in 2 mg/mL in methanol and working solutions were prepared by appropriate dilution of the standard stock solutions with methanol. All solutions were stored at −80 °C. Formic acid, ammonium acetate, acetonitrile, and methanol were purchased from Thermo Fisher Scientific Chemicals.

### 4.2. Sample Collection

Plasma, fecal, and urine samples from two human studies that included berry intake were used for qualitative analysis of BAs [48,49]. The studies were approved by the Institutional Review Board (Protocol#: IRB2015-083 and IRB2015030) of Illinois Institute of Technology (IIT), Chicago, Illinois. All subjects provided written informed consent before initiation of any study procedures. Whole blood samples were collected in ethylenediaminetetraacetic acid (EDTA) containing vacutainers and were centrifuged at 453× *g* for 15 min at 4 °C. Urine samples were collected in urine collection cups and then divided into 1 mL aliquots. Fecal samples were delivered in a cooler (with ice-packs) to the Clinical Nutrition Research Center within 24 h of collection and aliquoted into 50 mL falcon tubes prior storage. All biological samples were stored at −80 °C until analysis.

### 4.3. Sample Preparation

Plasma samples (500 μL) were thawed on ice and mixed with 1.5 mL water before extraction using solid-phase extraction (SPE) C18 cartridges (3 mL, 200 mg; Agilent Technologies, Santa Clara, CA, USA). SPE cartridges were conditioned with 2 mL of methanol followed by 2 mL of water. Diluted plasma samples were loaded into the cartridge. The SPE cartridges were then washed with 1 mL of water and eluted with 1 mL methanol. The eluent was subsequently dried under nitrogen gas and reconstituted in 100 μL of 50% methanol and centrifuged at 18,514× *g* for 10 min. The supernatant was transferred to auto-sampler vials for analysis.

Urine samples (500 μL) were thawed on ice and diluted with 1.5 mL water. The extraction of urine samples was the same as described above for plasma samples. The eluent from SPE cartridges was dried under nitrogen gas followed by reconstitution of the dried residue in 250 μL 50% methanol. The reconstituted sample was centrifuged at 18,514× *g* for 10 min and filtered through a 0.22 μm polytetrafluoroethylene (PTFE) syringe filter (Fisher Scientific Co., Pittsburg, PA, USA) before being transferred to auto-sampler vials.

Preparation of fecal samples was adapted from Amplatz et al. [18]. Briefly, fecal samples were freeze-dried and grinded thoroughly. Lyophilized fecal sample (100 mg) was incubated with 2 mL of NaOH (0.1 M) at 60 °C for 60 min in a water bath, followed by addition of water (4 mL). The mixture was vortexed for 30 s and then centrifuged at 18,514× *g* for 35 min. The collected supernatant was purified and concentrated using SPE tubes following the same method as described for plasma and urine.

### 4.4. Instrumentation

An Agilent 1290 Infinity ultra-high performance liquid chromatography (UHPLC) coupled with a 6550 iFunnel quadrupole time-of-flight (Q-TOF) with an electrospray ionization (ESI) source (Agilent Technologies, Santa Clara, CA, USA) was used for performing qualitative analysis. An Agilent 1290 Infinity UHPLC coupled with 6460 triple quadrupole (QQQ) with ESI was used for quantitative analysis. Chromatographic separations were achieved on an ACQUITY UHPLC BEH C18 column (1.7 μm, 2.1 × 100 mm) equipped with an ACQUITY BEH C18 1.7 μm VANGUARD Pre-Column (Waters, Milford, MA, USA) at 40 °C.

### 4.5. Liquid Chromatography and Mass Spectrometer Conditions

#### 4.5.1. Liquid Chromatographic Conditions

The chromatographic settings for qualitative analysis using UHPLC-Q-TOF were adapted from Yin et al. [36]. Mobile phase A (0.01% formic acid in water) and mobile phase B (acetonitrile) were used for the separation and elution of compounds with a flow rate of 0.35 mL/min. Gradient settings were as follows: 5% B (0.5 min), 35% B (1 min), 30% B (4 min), 30% B (5 min), 40% B (6 min), 45% B (8 min), 70% B (11.5 min), 100% B (12 min), 100% B (13 min), 5% B (13.1 min), 5% B (14 min) followed by a 5 min post-run. Chromatographic settings for quantitative analysis using UHPLC-QQQ were adapted from Bathena et al. [15]. The mobile phase A was ammonium acetate in water and mobile phase B consisted 20% acetonitrile in methanol with a flow rate of 0.25 mL/min. The gradient program was 55% B (12.75 min), 68% B (13 min), 68% B (22 min), 75% B (22.25 min), 75% B (29 min), 55% (29.25 min), followed by a 5-min post run to re-equilibrate the system. The injection volume was 5 μL for both qualitative and quantitative analysis.

To investigate the influence of ammonium acetate on the separation and detection of BAs using the UHPLC-QQQ system, BA standards mix solutions (1 μg/mL) were tested with 1 mM, 5 mM, 7.5 mM, and 10 mM ammonium acetate in mobile phase A, respectively; and 20% acetonitrile in methanol as mobile phase B. The pH of mobile phase A with different ammonium acetate concentrations was maintained within the range of 6.1–6.6. To investigate the influence of pH on the separation and detection of BAs using the UHPLC-QQQ system, BA standards mix solutions (1 μg/mL) were tested with mobile phase A containing 7.5 mM ammonium acetate in water, with pH adjusted to 3.8, 6.6, and 9.3 using acetic acid or ammonium hydroxide, and 20% acetonitrile in methanol as mobile phase B. All experiments were conducted in triplicates.

#### 4.5.2. Mass Spectrometric Conditions

The source parameters for ionization were as follows: gas temperature 250 °C, gas flow 10 L/min, nebulizer 35 psi, sheath gas heater 300 °C, sheath gas flow 11 L/min, capillary 2500 V, and VCharging 500. All BA species were detected and quantified in the negative ionization mode. Determination of precursor and product ion was based on the compound’s exact mass, fragmentation pattern, available reference standards, database search, and previous literature reports. The free BAs and their conjugated metabolites for which standards are not available were quantified using free BA standards with similar chemical properties and the parent BA standards, respectively. Collision energy for each compound was optimized by varying collision energy manually in a method, as well as using the MassHunter Optimizer software (Agilent Technologies).

### 4.6. Method Validation

The method for quantitation of BAs in human plasma using UHPLC-QQQ was developed and validated in terms of linearity, sensitivity, recovery, and matrix effect. Calibration curves were prepared in blank plasma following the same SPE procedures as used for human plasma sample preparation. A total of 20 BA standards were used in this study, out of which 16 BA standards were prepared in the range of 0.98 to 1000 ng/mL with 1 to 11 concentration levels, while GCDCA, GDCA, and DCA were prepared in range of 1.95 to 2000 ng/mL, and CA was prepared in range of 15.63 ng/mL to 16 μg/mL. All standards at different concentration levels were spiked with internal standards (GCDCA-D4, 500 ng/mL; CDCA-D4, 500 ng/mL). The limits of detection (LOD) and quantification (LOQ) were calculated based on a signal-to-noise ratio (S/N) of 3 for the LOD and of 10 for the LOQ.

Recoveries were evaluated by spiking a mixture of BA standards at a low (31.25 ng/mL) and a high concentration (1000 ng/mL) to blank plasma before sample preparation, followed by the same SPE procedures as for plasma sample preparation. Samples were prepared in triplicates. Recovery for each standard was calculated by average peak area of pre-spiked samples divided by average peak area of post-spiked samples at both low and high concentrations.

Matrix effect was evaluated for each standard by comparing the slopes of calibration curves in 50% methanol or in blank plasma after SPE procedure using the following equation: (slope in plasma–slope in solvent)/slope in solvent × 100%.

Precision was evaluated by injecting a mixture of BA standards (500 ng/mL), prepared in blank plasma, three times on a single day (intra-day precision) and on three different days (inter-day precision).

### 4.7. Clinical Trial Study Design

**Figure 7:**
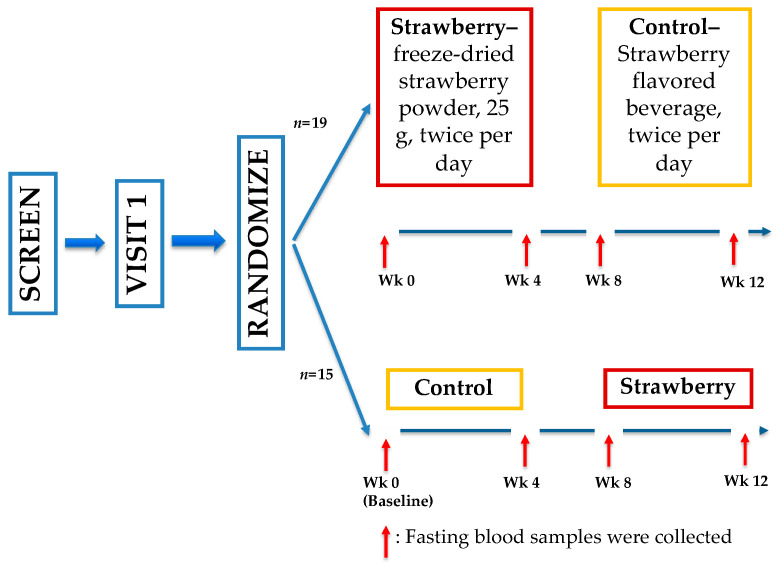
Study schema of the 4-week randomized, double-blind, placebo-controlled, crossover trial.

Plasma samples for quantitative analysis of BAs were used from a previous clinical trial (Figure 7) conducted by our group [50]. This study was approved by the Institutional Review Board (IRB2015-058) of Illinois Institute of Technology (IIT), Chicago, Illinois and registered with ClinicalTrials.gov (NCT 02612090). All subjects provided written informed consent before initiation of any study procedures. Briefly, 34 adults (age 52.6 ± 7.1 years, BMI 30.7 ± 3.6 kg m^−2^, mean ± standard deviation) were recruited for a four-week randomized, double-blind, placebo-controlled, crossover trial. Participants were randomized to 1 of 2 study sequences (control → strawberry or strawberry → control) with a four-week washout period between two intervention periods. During the intervention periods, participants consumed the strawberry beverage (25 g freeze-dried, equivalent to 1.75 cups of fresh strawberries) or the control beverage with matched calories twice a day, at least 6 h apart. Fasting blood samples were collected at weeks 0, 4, 8, and 12.

### 4.8. Statistical Analysis

Student’s *t*-test was used for statistical analysis for the comparison of different mobile phase conditions using Microsoft Excel (Version 16.41). Data were blinded for the statistical analysis of the clinical trial results. Normality tests were performed using Shapiro–Wilks test. Data not conforming to normal distribution were log10 transformed and indicated accordingly. Repeated measures analysis of variance using the Linear Mixed Models (SAS 9.4, SAS Institute, Inc., Cary NC, USA) were used for all chemical end-points assessing main effects of study beverages (baseline/week 0 vs. strawberries vs. control). When significant main effects were observed, post-hoc mean-separation testing was conducted using the Tukey–Kramer correction to adjust for multiple comparisons. Two-tailed *p* < 0.05 was considered significant after accounting for significant covariates (age, BMI, gender, and race). Results are presented as means ± standard errors of the mean.

## Figures and Tables

**Table 1:** Tentative identification of bile acid species in human biological samples using UHPLC-Q-TOF.

Compounds	RT (min)	UHPLC-Q-TOF Qualification					Reference(s)
		*m*/*z*^1^	MS/MS Fragments ^2^	Occurrence ^3^			
3-oxo-LCA-isomer 1	12.5	373.2728	373.2728		F		[13]
3-oxo-LCA-isomer 2	12.9	373.2745	373.2745		F		[13]
isoLCA	12.3	375.2899	375.2899		F		[13]
LCA	12.8	375.2906	375.2906		F		Standard
Nor-DCA	13.0	377.2685	377.2685	P	F	U	[13]
5α-cholanic acid-3,6-dione isomer 1	10.2	387.2544	387.2544		F		[13]
5α-cholanic acid-3,6-dione isomer 2	10.5	387.2542	387.2542		F		[13]
5α-cholanic acid-3,6-dione isomer 3	11.1	387.2524	387.2524		F		[13]
7-oxo-LCA isomer 1	9.1	389.2696	389.2696		F		[13]
7-oxo-LCA isomer 2	9.5	389.2692	389.2692		F		[13]
7-oxo-LCA isomer 3	9.8	389.2683	389.2683		F		[13]
7-oxo-LCA isomer 4	10.1	389.2699	389.2699		F		[13]
7-oxo-LCA isomer 5	10.4	389.2698	389.2698		F		[13]
7-oxo-LCA isomer 6	11.1	389.2683	389.2683		F		[13]
7-oxo-LCA isomer 7	13.0	389.2701	389.2701		F		[13]
MuriDCA	8.3	391.2842	391.2842	P	F		Standard
UDCA	8.9	391.2845	391.2845	P	F		Standard
HDCA isomer	9.7	391.2853	391.2853	P	F	U	[13]
CDCA	10.8	391.2850	391.2850	P	F		Standard
DCA	11.1	391.2846	391.2846	P	F	U	Standard
3α-hydroxy-6,7-diketocholanic acid	6.2	403.2479	403.2479			U	[13]
7-oxo-DCA isomer 1	4.8	405.2644	405.2644		F		[13]
7-oxo-DCA isomer 2	7.2	405.2639	405.2639		F		[13]
7-oxo-DCA isomer 3	7.8	405.2634	405.2634		F		[13]
β -MCA isomer 1	3.1	407.2809	407.2809		F		[13]
β -MCA isomer 2	4.5	407.2804	407.2804		F	U	[13]
β -MCA isomer 3	4.9	407.2808	407.2808		F	U	[13]
β -MCA isomer 4	5.3	407.2807	407.2807		F		[13]
β -MCA isomer 5	6.7	407.2805	407.2805		F		[13]
β-MCA	7.3	407.2797	407.2797		F		Standard
HCA isomer 1	5.9	407.2814	407.2814		F		[13]
HCA isomer 2	8.1	407.2793	407.2793		F		[13]
HCA isomer 3	8.4	407.2806	407.2806		F		[13]
CA	8.7	407.2809	407.2809	P	F		Standard
GLCA	11.1	432.3121	74.0242	P			Standard
Glyco-7-oxo-LCA isomer 1	4.6	446.2900	74.0247	P		U	No reference ^4^
Glyco-7-oxo-LCA isomer 2	5.6	446.2910	74.0239	P			No reference
Glyco-7-oxo-LCA isomer 3	7.9	446.2894	74.0243	P	F	U	No reference
GUDCA isomer 1	6.7	448.3060	74.0244	P	F		Standard
GUDCA isomer 2	7.6	448.3068	74.0243	P	F	U	Standard
GCDCA	8.9	448.3059	74.0242	P	F		Standard
GDCA	9.3	448.3066	74.0246	P	F		Standard
3-oxo-LCA-sulfate isomer	8.1	453.2292	96.9599		F		[13]
LCA-sulfate	8.3	455.2478	96.9598		F		[13]
Glyco-12-oxo-CDCA isomer	3.9	462.2860	74.0239	P			No reference
GHCA isomer 1	3.0	464.3006	74.0240	P		U	[13]
GHCA isomer 2	3.4	464.3001	74.0245	P		U	[13]
GHCA	4.7	464.3006	74.0242	P		U	[13]
GCA	6.9	464.3023	74.0244	P	F		Standard
12-oxo-LCA-sulfate	3.3	469.2265	96.9599		F		[13]
CDCA-sulfate isomer 1	3.8	471.2426	96.9598		F		[13]
CDCA-sulfate isomer 2	6.5	471.2433	96.9597		F	U	[13]
CDCA-sulfate isomer 3	7.5	471.2425	96.9598	P	F	U	[13]
12-oxo-CDCA sulfate isomer	2.6	485.2220	96.9598		F		[13]
CA-sulfate isomer 1	2.8	487.2377	96.9597		F		[13]
CA-sulfate isomer 2	3.5	487.2368	96.9595		F		[13]
TCDCA isomer 1	7.0	498.2896	79.9571	P			Standard
TCDCA isomer 2	7.7	498.2892	79.9567	P			Standard
TCDCA isomer 3	8.2	498.2889	79.9565	P			Standard
TCA isomer 1	2.3	514.2837	514.2837 79.9566	P	F		[13,27]
TCA isomer 2	3.2	514.2844	514.2844 79.9573	P			[13,27]
GCDCA-sulfate isomer 1	3.2	528.2627	74.0242 96.9597			U	[13]
GCDCA-sulfate isomer 2	7.0	528.2627	74.0245 96.9598			U	[13]
GCA-sulfate isomer	3.4	544.2591	96.9596 464.3014			U	[28]
Glucuronide-12-oxo-LCA isomer 1	5.4	565.3009	389.2712 75.0084			U	[27]
Glucuronide-12-oxo-LCA isomer 2	5.8	565.2995	389.2685 75.0080	P		U	[27]
Glucuronide-12-oxo-LCA isomer 3	6.4	565.3021	389.2704 75.0082	P			[27]
Glucuronide-CDCA isomer 1	4.2	567.3178	391.2847 75.0077			U	[27,29]
Glucuronide-CDCA isomer 2	6.7	567.3168	391.2858 75.0078	P		U	[27,29]
Glucuronide-CDCA isomer 3	8.5	567.3175	391.2845 75.0080	P		U	[27,29]
Glucuronide-CA isomer 1	2.6	583.3137	407.2799 75.0081	P		U	[27,29]
Glucuronide-CA isomer 2	4.0	583.3130	407.2804 75.0085			U	[27,29]
Glucuronide-CA isomer 3	5.3	583.3117	407.2800 75.0086	P		U	[27,29]
Glucuronide-CA isomer 4	5.9	583.3100	407.2798 75.0071			U	[27,29]
Glucuronide-GLCA	8.6	608.3425	432.3117 74.0242	P			No reference
Glucuronide-GCDCA isomer 1	3.0	624.3400	448.3051 74.0245	P		U	No reference
Glucuronide-GCDCA isomer 2	7.0	624.3388	448.3070 74.0246	P		U	No reference
Glucuronide-GCDCA isomer 3	7.4	624.3385	448.3062 74.0245			U	No reference
Glucuronide-GCA isomer 1	2.2	640.3318	464.3014 74.0234	P		U	No reference
Glucuronide-GCA isomer 2	2.9	640.3344	464.3017 74.0240			U	No reference
Glucuronide-GCA isomer 3	3.3	640.3345	464.3019 74.0239	P			No reference

^1^*m*/*z* corresponds to [M-H]^1−^. ^2^ MS/MS fragments: MS/MS fragments listed as Quantifier/Qualifier ion used in the UHPLC-QQQ method. Single value means the same ion was used as both quantifier and qualifier ion. ^3^ Detected in P, plasma; U, urine; F, feces. ^4^ No reference available, MS/MS spectrum attached in the Supplementary file Table S1. Abbreviations: cholic acid (CA), chenodeoxycholic acid (CDCA), deoxycholic acid (DCA), glycocholic acid (GCA), glycochenodeoxycholic acid (GCDCA), glycodeoxycholic acid (GDCA), glycolithocholic acid (GLCA), glycoursodeoxycholic acid (GUDCA), hyocholic acid (HCA), hyodeoxycholic acid (HDCA), lithocholic acid (LCA), β-muricholic acid (β-MCA), murideoxycholic acid (MuriDCA), taurocholic acid (TCA), taurochenodeoxycholic acid (TCDCA), taurodeoxycholic acid (TDCA), taurolithochoic acid (TLCA), tauroursodeoxycholic acid (TUDCA), taurohyodeoxycholic acid (THDCA), ursodeoxycholic acid (UDCA).

**Table 2:** Linearity range, LOQ, LOD, recovery, matrix effect, and precision of bile acids in plasma—UHPLC-QQQ method validation ^1^.

Compounds	Linearity Range nmol/L	Correlation Coefficient (R^2^)	LOQ ^2^ nmol/L	LOD ^3^ nmol/L	%Recovery at High Level ^4^	%Recovery at Low Level ^4^	%Matrix Effect	Intra-Day Precision (RSD%) ^5^	Inter-Day Precision (RSD%) ^5^
TUDCA	LOQ–2002.9	0.9977	2.5	0.3	105 ± 2	82 ± 4	5	2.2	14.7
THDCA	LOQ–2002.9	0.9986	2.5	0.2	105 ± 3	79 ± 1	8	1.6	13.0
TCDCA	LOQ–2002.9	0.9914	2.5	0.6	136 ± 9	104 ± 6	−5	1.2	8.2
TDCA	LOQ–2002.9	0.9927	2.5	0.6	112 ± 3	90 ± 9	−3	0.7	8.0
GUDCA	LOQ–2225.6	0.9999	2.8	0.7	117 ± 5	99 ± 3	16	3.2	8.9
GCDCA	LOQ–2225.6	0.9999	2.8	0.4	114 ± 3	105 ± 8	10	1.5	5.4
GDCA	LOQ–4451.3	0.9997	2.8	0.4	112 ± 3	107 ± 4	−2	0.4	4.4
TCA	LOQ–1940.6	0.9988	4.9	0.3	123 ± 6	90 ± 2	6	5.2	10.8
TLCA	LOQ–2069.2	0.9994	2.6	0.7	114 ± 5	89 ± 6	−14	4.5	5.1
GCA	LOQ–2149.2	0.9994	5.4	1.3	116 ± 8	91 ± 9	31	3.5	11.4
GLCA	LOQ–2307.9	0.9998	1.4	0.2	106 ± 9	103 ± 4	−5	3.0	7.5
β-MCA	LOQ–2447.4	0.9996	6.1	3.1	112 ± 3	93 ± 4	14	0.4	3.1
HCA	LOQ–2447.4	0.9995	6.1	3.1	77 ± 9	80 ± 17	23	2.8	4.9
CA	LOQ–39158.1	0.9979	49.0	6.1	123 ± 5	90 ± 6	7	3.8	6.7
MuriDCA	LOQ–2549.1	0.9974	6.4	3.2	115 ± 4	99 ± 6	14	0.9	4.9
UDCA	LOQ–2549.1	0.9995	6.4	3.2	117 ± 3	100 ± 3	0	2.2	2.9
HDCA	LOQ–2549.1	0.9995	6.4	3.2	84 ± 15	74 ± 13	0	1.5	11.2
CDCA	LOQ–2549.1	0.9988	6.4	3.2	105 ± 7	84 ± 3	−11	0.3	0.9
DCA	LOQ–5098.1	0.9975	6.4	1.6	151 ± 6	94 ± 4	−11	0.6	7.1
LCA	LOQ–2657.5	0.9994	6.6	3.3	98 ± 11	84 ± 4	−17	2.1	4.1

^1^ Recovery for each standard was calculated using the equation: average peak area of pre-spiked samples/average peak area of post-spiked samples; matrix effect was calculated using the equation: (slope in plasma–slope in solvent)/slope in solvent × 100. ^2^ LOQ: limit of quantification. ^3^ LOD: limit of detection. ^4^ Data are presented as mean ± standard deviation. ^5^ Relative standard deviations (RSD %) calculated with the standards in blank plasma.

**Table 3:** Bile acids (BAs) concentrations in fasting human plasma samples after 4-week strawberry or control beverage intake.

Compounds	Transition (*m*/*z*)	Quantifier/Qualifier Ion (*m*/*z*) ^1^	Retention Time (min)	Baseline ^2,3^ (μmol/L)	Control ^3^ (μmol/L)	STR ^3,4^ (μmol/L)	*p*-Value			
							Treatment Effect	Baseline vs. Control	Baseline vs. STR	Control vs. STR
CA	407.30	407.30	9.47	0.70 ± 0.51	1.18 ± 1.05	0.25 ± 0.10	NS ^5^	NS	NS	NS
GCA	464.30	73.90	7.23	1.22 ± 0.46	2.00 ± 1.03	0.82 ± 0.17	NS	NS	NS	NS
GCDCA	448.31	74.02	14.86	5.30 ± 1.61	7.54 ± 3.01	3.44 ± 0.62	NS	NS	NS	NS
CDCA	391.30	391.30	16.27	1.51 ± 0.41	2.20 ± 1.25	1.18 ± 0.32	NS	NS	NS	NS
TCA	514.30	514.30	7.31	0.29 ± 0.10	0.41 ± 0.25	0.18 ± 0.04	NS	NS	NS	NS
TCDCA	498.29	79.96	14.94	0.59 ± 0.20	0.63 ± 0.33	0.35 ± 0.07	NS	NS	NS	NS
**Total Primary**				9.60 ± 2.55	13.96 ± 5.14	6.21 ± 0.99	NS	NS	NS	NS
beta-MCA	407.30	407.30	2.30	0.02 ± 0.00	0.03 ± 0.00	0.02 ± 0.00	NS	NS	NS	NS
beta-MCA isomer	407.30	407.30	3.04	0.01 ± 0.00	0.03 ± 0.01	0.02 ± 0.01	0.03 ^6^	0.03	NS	NS
HCA	407.30	407.30	5.22	0.03 ± 0.01	0.03 ± 0.01	0.04 ± 0.01	NS	NS	NS	NS
HCA isomer	407.30	407.30	6.62	0.02 ± 0.01	0.03 ± 0.01	0.02 ± 0.01	NS	NS	NS	NS
GLCA	432.30	73.90	18.58	0.33 ± 0.08	0.62 ± 0.19	0.29 ± 0.06	0.02	NS	NS	0.04
Glucuronide-GLCA	608.34	432.31/74.02	4.53	0.24 ± 0.06	0.44 ± 0.13	0.20 ± 0.04	0.02	NS	NS	0.04
GLCA-sulfate	512.27	74.02	5.72	0.10 ± 0.02	0.13 ± 0.02	0.09 ± 0.01	0.04	NS	NS	NS
GUDCA	448.31	74.02	4.30	0.68 ± 0.15	1.25 ± 0.39	0.54 ± 0.13	<0.01	0.05	NS	<0.01
GDCA	448.31	74.02	14.86	4.03 ± 1.24	5.42 ± 1.48	2.47 ± 0.32	0.03	NS	NS	0.04
LCA	375.30	375.30	21.86	0.04 ± 0.01	0.05 ± 0.01	0.03 ± 0.01	0.04	NS	NS	0.04
LCA-sulfate isomer 1	455.25	96.96	5.84	0.01 ± 0.00	0.01 ± 0.00	0.01 ± 0.00	NS	NS	NS	NS
LCA-sulfate isomer 2	455.25	96.96	2.07	<LOQ ^7^	<LOQ	<LOQ	NA ^8^	NA	NA	NA
LCA-sulfate isomer 3	455.25	96.96	4.21	<LOQ	<LOQ	<LOQ	NA	NA	NA	NA
LCA-sulfate isomer 4	455.25	96.96	10.71	<LOQ	<LOQ	<LOQ	NA	NA	NA	NA
LCA-sulfate isomer 5	455.25	96.96	4.52	<LOQ	<LOQ	<LOQ	NA	NA	NA	NA
3-OXO-LCA isomer 1	373.27	373.27	14.94	0.01 ± 0.00	0.01 ± 0.00	0.01 ± 0.00	NS	NS	NS	NS
3-OXO-LCA isomer 2	373.27	373.27	3.35	0.00 ± 0.00	0.00 ± 0.00	0.00 ± 0.00	NS	NS	NS	NS
3-OXO-LCA isomer 3	373.27	373.27	12.54	<LOQ	<LOQ	0.00 ± 0.00	NA	NA	NA	NA
3-OXO-LCA isomer 4	373.27	373.27	19.64	0.00 ± 0.00	0.00 ± 0.00	0.00 ± 0.00	NA	NA	NA	NA
Glyco-3-OXO-LCA isomer 1	430.30	74.02	8.90	0.00 ± 0.00	0.00 ± 0.00	0.00 ± 0.00	0.03	NS	NS	NS
Glyco-3-OXO-LCA isomer 2	430.30	74.02	18.44	<LOQ	<LOQ	<LOQ	NA	NA	NA	NA
Glyco-3-OXO-LCA isomer 3	430.30	74.02	23.35	0.00 ± 0.00	0.00 ± 0.00	<LOQ	<0.01	<0.01	NA	NA
3-OXO-LCA-sulfate isomer 1	453.23	96.96	2.95	<LOQ	<LOQ	<LOQ	NA	NA	NA	NA
3-OXO-LCA-sulfate isomer 2	453.23	96.96	3.35	<LOQ	<LOQ	<LOQ	NA	NA	NA	NA
3-OXO-LCA-sulfate isomer 3	453.23	96.96	4.32	<LOQ	<LOQ	<LOQ	NA	NA	NA	NA
3-OXO-LCA-sulfate isomer 4	453.23	96.96	5.92	<LOQ	<LOQ	<LOQ	NA	NA	NA	NA
3-OXO-LCA-sulfate isomer 5	453.23	96.96	15.74	<LOQ	<LOQ	<LOQ	NA	NA	NA	NA
3-OXO-LCA-sulfate isomer 6	453.23	96.96	16.02	<LOQ	<LOQ	<LOQ	NA	NA	NA	NA
7-OXO-LCA isomer 1	389.27	389.27	14.88	0.05 ± 0.01	0.07 ± 0.02	0.04 ± 0.01	NS	NS	NS	NS
7-OXO-LCA isomer 2	389.27	389.27	3.03	0.00 ± 0.00	0.00 ± 0.00	0.00 ± 0.00	NS	NS	NS	NS
7-OXO-LCA isomer 3	389.27	389.27	3.58	0.03 ± 0.01	0.04 ± 0.01	0.03 ± 0.01	NS	NS	NS	NS
7-OXO-LCA isomer 4	389.27	389.27	3.85	0.05 ± 0.01	0.07 ± 0.01	0.06 ± 0.01	0.01	0.01	NS	NS
7-OXO-LCA isomer 5	389.27	389.27	5.20	0.01 ± 0.00	0.02 ± 0.01	0.01 ± 0.00	NS	NS	NS	NS
7-OXO-LCA isomer 6	389.27	389.27	7.70	0.01 ± 0.00	0.01 ± 0.00	0.01 ± 0.01	NS	NS	NS	NS
7-OXO-LCA isomer 7	389.27	389.27	8.47	0.02 ± 0.01	0.02 ± 0.01	0.01 ± 0.00	NS	NS	NS	NS
7-OXO-LCA isomer 8	389.27	389.27	16.56	<LOQ	<LOQ	<LOQ	NA	NA	NA	NA
Glyco-7-oxo-LCA isomer 1	446.29	74.02	6.26	0.02 ± 0.01	0.04 ± 0.02	0.01 ± 0.00	NS	NS	NS	NS
Glyco-7-oxo-LCA isomer 2	446.29	74.02	2.46	0.01 ± 0.00	0.00 ± 0.00	0.00 ± 0.00	NS	NS	NS	NS
Glyco-7-oxo-LCA isomer 3	446.29	74.02	3.63	<LOQ	0.00 ± 0.00	<LOQ	NA	NA	NA	NA
Glyco-7-oxo-LCA isomer 4	446.29	74.02	6.55	0.01 ± 0.01	0.03 ± 0.02	0.01 ± 0.00	NS	NS	NS	NS
Glucuronide-12-oxo-LCA isomer 1	565.30	389.27/ 75.01	1.88	<LOQ	<LOQ	<LOQ	NA	NA	NA	NA
Glucuronide-12-oxo-LCA isomer 2	565.30	389.27/ 75.01	2.17	<LOQ	<LOQ	<LOQ	NA	NA	NA	NA
Glucuronide-12-oxo-LCA isomer 3	565.30	389.27/ 75.01	4.04	<LOQ	<LOQ	<LOQ	NA	NA	NA	NA
3α-hydroxy-6,7-diketo cholanic acid isomer 1	403.25	403.25	3.60	0.00 ± 0.00	0.00 ± 0.00	0.00 ± 0.00	NS	NS	NS	NS
3α-hydroxy-6,7-diketo cholanic acid isomer 2	403.25	403.25	4.33	0.01 ± 0.00	0.00 ± 0.00	<LOQ	NS	NS	NS	NS
3α-hydroxy-6,7-diketo cholanic acid isomer 3	403.25	403.25	1.40	<LOQ	0.00 ± 0.00	0.00 ± 0.00	NA	NA	NA	NA
5α-cholanic acid-3,6-dione isomer 1	387.25	387.25	6.02	0.02 ± 0.00	0.04 ± 0.01	0.04 ± 0.01	<0.01	<0.01	0.01	NS
5α-cholanic acid-3,6-dione isomer 2	387.25	387.25	1.40	0.01 ± 0.00	0.01 ± 0.00	0.01 ± 0.00	NS	NS	NS	NS
5α-cholanic acid-3,6-dione isomer 3	387.25	387.25	15.62	<LOQ	0.00 ± 0.00	0.00 ± 0.00	NS	NS	NS	NS
5α-cholanic acid-3,6-dione isomer 4	387.25	387.25	19.30	0.02 ± 0.01	0.02 ± 0.01	0.01 ± 0.00	NS	NS	NS	NS
5α-cholanic acid-3,6-dione isomer 5	387.25	387.25	1.80	0.02 ± 0.01	0.03 ± 0.01	0.03 ± 0.01	NS	NS	NS	NS
MuriDCA	391.30	391.30	3.78	1.20 ± 0.36	1.84 ± 0.55	1.61 ± 0.47	NS	NS	NS	NS
UDCA	391.30	391.30	5.88	0.53 ± 0.11	0.75 ± 0.14	0.57 ± 0.12	NS	NS	NS	NS
HDCA	391.30	391.30	7.41	0.93 ± 0.16	1.31 ± 0.29	1.16 ± 0.26	NS	NS	NS	NS
DCA	391.30	391.30	16.95	3.33 ± 0.56	4.48 ± 1.00	2.69 ± 0.33	0.01	NS	NS	0.01
nor-DCA isomer 1	377.27	377.27	7.70	0.05 ± 0.01	0.05 ± 0.01	0.04 ± 0.01	NS	NS	NS	NS
nor-DCA isomer 2	377.27	377.27	10.39	0.02 ± 0.00	0.02 ± 0.01	0.02 ± 0.01	NS	NS	NS	0.05
nor-DCA isomer 3	377.27	377.27	1.33	0.02 ± 0.01	0.02 ± 0.01	0.02 ± 0.01	NS	NS	NS	NS
nor-DCA isomer 4	377.27	377.27	16.43	0.02 ± 0.01	0.01 ± 0.00	0.01 ± 0.00	NS	NS	NS	NS
nor-DCA isomer 5	377.27	377.27	18.17	0.02 ± 0.01	0.02 ± 0.01	0.02 ± 0.01	0.05	NS	0.04	NS
7-oxo-DCA isomer 1	405.26	96.96	1.75	0.02 ± 0.01	0.02 ± 0.01	0.02 ± 0.01	NS	NS	NS	NS
12-oxo-CDCA-sulfate isomer 1	485.22	96.96	4.70	0.00 ± 0.00	<LOQ	<LOQ	NA	NA	NA	NA
12-oxo-CDCA-sulfate isomer 2	485.22	96.96	13.98	0.00 ± 0.00	0.01 ± 0.00	0.00 ± 0.00	<0.01	<0.01	<0.01	<0.01
Glyco-12-oxo-CDCA isomer 1	462.29	74.02	2.08	0.01 ± 0.00	0.02 ± 0.00	0.01 ± 0.00	NS	NS	NS	NS
Glyco-12-oxo-CDCA isomer 2	462.29	74.02	2.55	0.01 ± 0.00	0.01 ± 0.00	0.01 ± 0.00	NS	NS	NS	NS
Glyco-12-oxo-CDCA isomer 3	462.29	74.02	1.59	0.00 ± 0.00	0.01 ± 0.00	0.01 ± 0.00	NA	NA	NA	NA
Glyco-12-oxo-CDCA isomer 4	462.29	74.02	2.90	0.01 ± 0.00	0.01 ± 0.00	0.01 ± 0.00	NS	NS	NS	NS
TCA isomer 1	514.30	514.30/79.96	4.21	0.03 ± 0.01	0.03 ± 0.01	0.02 ± 0.00	NS	NS	NS	NS
TCA isomer 2	514.30	514.30/79.96	14.86	0.03 ± 0.00	0.03 ± 0.00	0.03 ± 0.00	NS	NS	NS	NS
TCA isomer 3	514.30	514.30/79.96	15.56	0.03 ± 0.00	0.03 ± 0.00	0.03 ± 0.00	NS	NS	NS	NS
TLCA	482.29	482.29	18.54	0.01 ± 0.00	0.03 ± 0.02	0.01 ± 0.00	0.02	0.02	NS	NS
TLCA-sulfate	562.25	482.29	5.88	0.00 ± 0.00	0.00 ± 0.00	0.00 ± 0.00	NS	NS	NS	NS
TUDCA	498.29	79.96	4.40	0.01 ± 0.00	0.02 ± 0.01	0.01 ± 0.00	NS	NS	NS	NS
THDCA	498.29	79.96	5.14	0.01 ± 0.00	0.01 ± 0.01	0.00 ± 0.00	NS	NS	NS	NS
TDCA	498.29	79.96	15.60	0.47 ± 0.12	0.80 ± 0.46	0.27 ± 0.06	NS	NS	NS	NS
**Total Secondary**				12.73 ± 1.92	18.14 ± 2.98	10.74 ± 1.07	<0.01	< 0.01	NS	<0.01
Glucuronide-GCDCA isomer 1	624.34	448.31/74.02	2.67	0.26 ± 0.08	0.26 ± 0.05	0.19 ± 0.04	NS	NS	NS	NS
Glucuronide-GCDCA isomer 2	624.34	448.31/74.02	3.05	0.11 ± 0.04	0.12 ± 0.05	0.13 ± 0.07	NS	NS	NS	NS
GCDCA-sulfate isomer 1	528.26	74.02/96.96	2.95	0.01 ± 0.00	0.01 ± 0.00	0.01 ± 0.00	0.01	0.01	NS	NS
GCDCA-sulfate isomer 2	528.26	74.02/96.96	3.33	0.03 ± 0.01	0.03 ± 0.02	0.01 ± 0.00	NS	NS	NS	NS
GCDCA-sulfate isomer 3	528.26	74.02/96.96	6.24	0.00 ± 0.00	0.00 ± 0.00	0.00 ± 0.00	NS	NS	NS	NS
Glucuronide-CA isomer 1	583.31	407.27/75.01	1.69	0.05 ± 0.02	0.07 ± 0.02	0.05 ± 0.02	NS	NS	NS	NS
Glucuronide-CA isomer 2	583.31	407.27/75.01	2.26	0.02 ± 0.01	0.02 ± 0.01	0.02 ± 0.01	NS	NS	NS	NS
CA-sulfate isomer 1	487.24	96.96	1.39	0.03 ± 0.01	0.04 ± 0.02	0.03 ± 0.01	NS	NS	NS	NS
CA-sulfate isomer 2	487.24	96.96	2.87	0.07 ± 0.02	0.08 ± 0.02	0.14 ± 0.07	NS	NS	NS	NS
Glucuronide-GCA	640.33	464.30/74.02	1.85	0.03 ± 0.01	0.02 ± 0.01	0.02 ± 0.01	NS	NS	NS	NS
Glucuronide-CDCA isomer 1	567.32	391.28/75.01	1.79	0.02 ± 0.00	0.02 ± 0.00	0.02 ± 0.00	NS	NS	NS	NS
Glucuronide-CDCA isomer 2	567.32	391.28/75.01	2.12	0.02 ± 0.00	0.02 ± 0.01	0.02 ± 0.00	NS	NS	NS	NS
Glucuronide-CDCA isomer 3	567.32	391.28/75.01	2.56	0.00 ± 0.00	0.01 ± 0.00	0.00 ± 0.00	NS	NS	NS	NS
Glucuronide-CDCA isomer 4	567.32	391.28/75.01	4.00	0.01 ± 0.00	0.02 ± 0.01	0.02 ± 0.01	NS	NS	NS	NS
Glucuronide-CDCA isomer 5	567.32	391.28/75.01	5.31	0.01 ± 0.00	0.01 ± 0.00	0.01 ± 0.00	NS	NS	NS	NS
CDCA-sulfate isomer 1	471.24	96.96	1.83	0.02 ± 0.01	0.02 ± 0.01	0.02 ± 0.01	NS	NS	NS	NS
CDCA-sulfate isomer 2	471.24	96.96	2.81	0.03 ± 0.01	0.04 ± 0.01	0.03 ± 0.01	NS	NS	NS	NS
CDCA-sulfate isomer 3	471.24	96.96	4.82	0.01 ± 0.00	0.01 ± 0.00	0.01 ± 0.00	NS	NS	NS	NS
CDCA-sulfate isomer 4	471.24	96.96	5.39	0.01 ± 0.00	0.01 ± 0.00	0.01 ± 0.00	NS	NS	NS	NS

^1^ MS/MS fragments listed as Quantifier/Qualifier ion. Single ion transition means the same ion was used as both quantifier and qualifier ion; ^2^ Baseline BAs concentrations used data from Week 0. ^3^ Data are presented as mean ± standard error of the mean; Used 0.00 when value is <0.001; ^4^ STR: Strawberry; ^5^ NS: Not significant (*p* > 0.05); ^6^ *p* values are presented as the exact value rounded to 2 decimals, *p* ≤ 0.05 shown in this table was considered significant; ^7^ LOQ: limit of quantification; ^8^ NA: data not available due to lower than limit of quantification or limited number of observations in subjects.

## Data Availability

The data presented in this study are available in this article and supplementary material. Raw data are available on request.

## Supplementary Materials

The following are available online at https://www.mdpi.com/2218-1989/11/2/99/s1, Table S1: UHPLC-Q-TOF chromatograms of tentatively identified BAs in human plasma, feces, and urine samples, Table S2: BA standards used for quantification of individual BA metabolites, Table S3: Nutritional composition of control and strawberry beverage (per 50 g portion).
