# Supplementary material for: Comprehensive Characterization of Bile Acids in Human Biological Samples and Effect of 4-Week Strawberry Intake on Bile Acid Composition in Human Plasma
Source: Metabolites. 2021 Feb 10;11(2):99. doi: 10.3390/metabo11020099 (PMC7916557; doi:10.3390/metabo11020099)
Supplement: Supplementary file 1 [file metabolites-11-00099-s001.pdf]

Supplementary Material

Table S1. UHPLC-Q-TOF chromatograms of tentatively identified BAs in human plasma, feces and urine samples.

Compounds	RT (min)	UHPLC-Q-TOF Qualification			UHPLC-Q-TOF Chromatograms	Reference/s
		Observed (m/z)[1]	MS/MS fragments (Quantifier/ Qualifier ion) (m/z)[2]	Occurrence[3]		
3-oxo-LCA-isomer 1	12.5	373.2728	373.2728	F	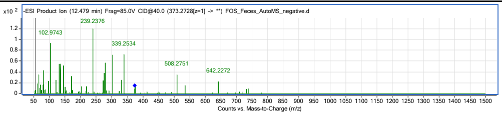	[13]
3-oxo-LCA-isomer 2	12.9	373.2745	373.2745	F	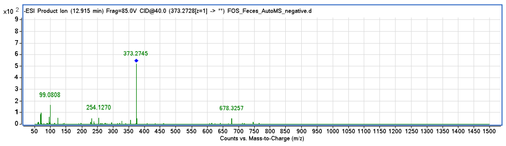	[13]
isoLCA	12.3	375.2899	375.2899	F	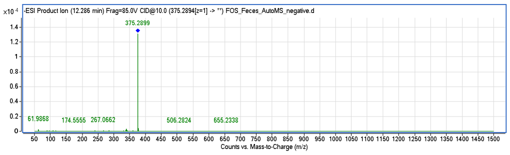	[13]
LCA	12.8	375.2906	375.2906	F	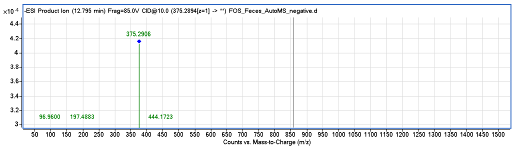	Standard
Nor-DCA	13.0	377.2685	377.2685	P F U	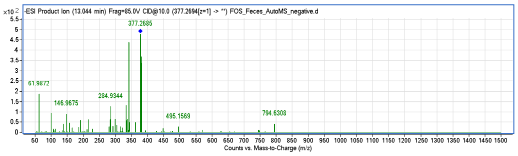	[13]
5α-cholanic acid-3,6-dione isomer 1	10.2	387.2544	387.2544	F	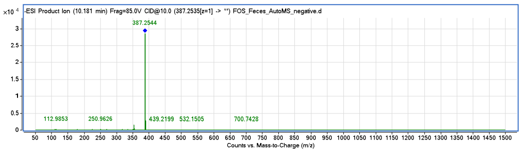	[13]
5α-cholanic acid-3,6-dione isomer 2	10.5	387.2542	387.2542	F	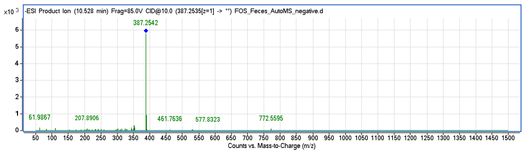	[13]

5α-cholanic acid-3,6-dione isomer 3	11.1	387.2524	387.2524		F	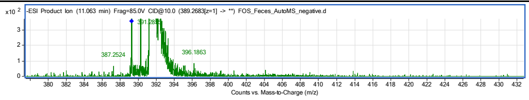	[13]
7-oxo-LCA isomer 1	9.1	389.2696	389.2696		F	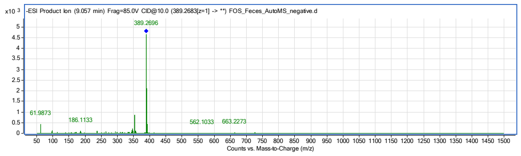	[13]
7-oxo-LCA isomer 2	9.5	389.2692	389.2692		F	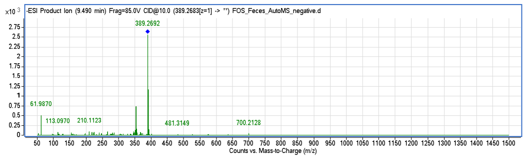	[13]
7-oxo-LCA isomer 3	9.8	389.2683	389.2683		F	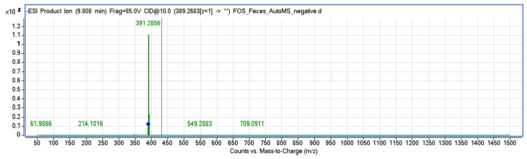	[13]
7-oxo-LCA isomer 4	10.1	389.2699	389.2699		F	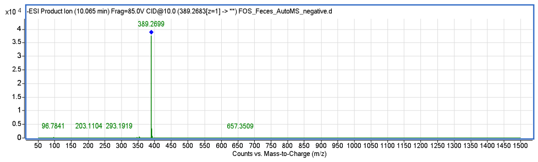	[13]
7-oxo-LCA isomer 5	10.4	389.2698	389.2698		F	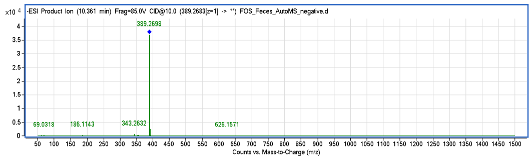	[13]
7-oxo-LCA isomer 6	11.1	389.2683	389.2683		F	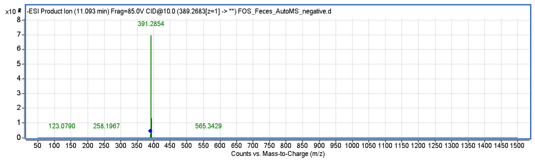	[13]
7-oxo-LCA isomer 7	13.0	389.2701	389.2701		F	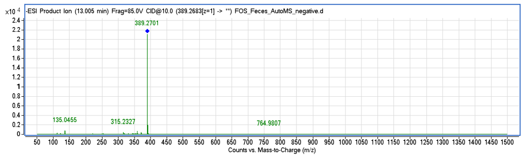	[13]
MuriDCA	8.3	391.2842	391.2842	P	F	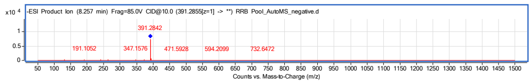	Standard
UDCA	8.9	391.2845	391.2845	P	F	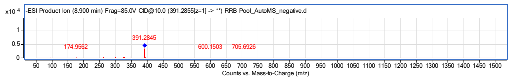	Standard

HDCA isomer	9.7	391.2853	391.2853	P	F	U	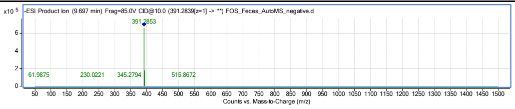	[13]
CDCA	10.8	391.2850	391.2850	P	F		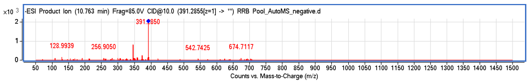	Standard
DCA	11.1	391.2846	391.2846	P	F	U	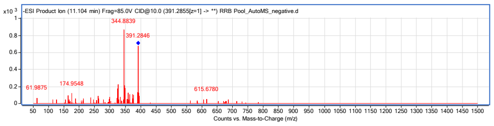	Standard
3α-hydroxy-6,7-diketocholanic acid	6.2	403.2479	403.2479			U	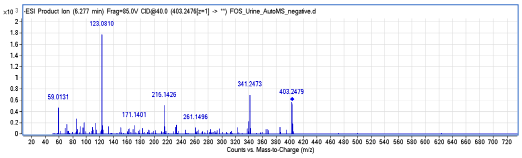	[13]
7-oxo-DCA isomer 1	4.8	405.2644	405.2644		F		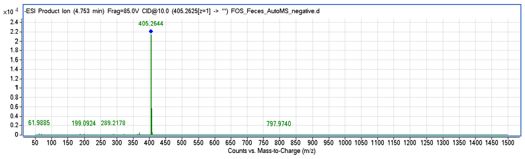	[13]
7-oxo-DCA isomer 2	7.2	405.2639	405.2639		F		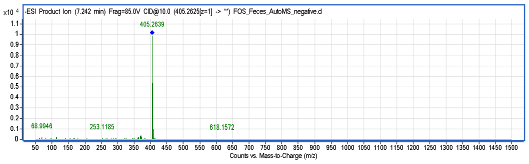	[13]
7-oxo-DCA isomer 3	7.8	405.2634	405.2634		F		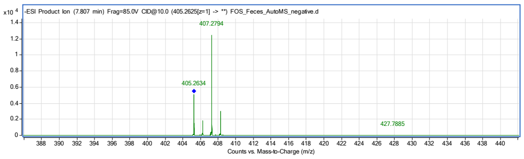	[13]
β -MCA isomer 1	3.1	407.2809	407.2809		F		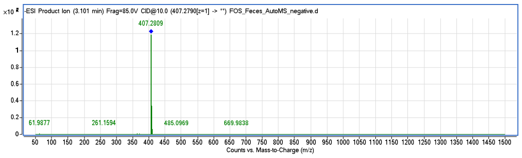	[13]
β -MCA isomer 2	4.5	407.2804	407.2804		F	U	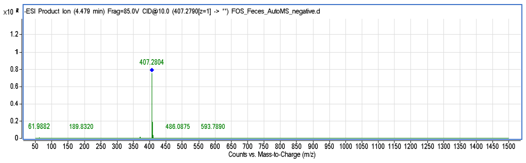	[13]
β -MCA isomer 3	4.9	407.2808	407.2808		F	U	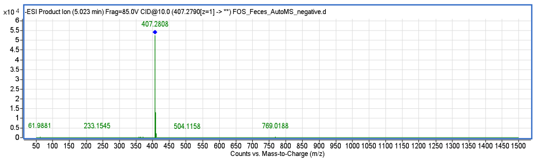	[13]

β -MCA isomer 4	5.3	407.2807	407.2807		F		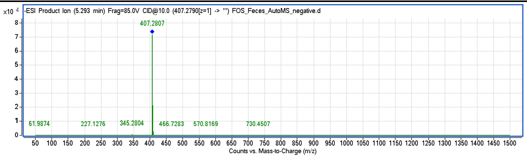	[13]
β -MCA isomer 5	6.7	407.2805	407.2805		F		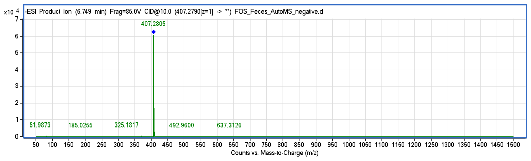	[13]
β-MCA	7.3	407.2797	407.2797		F		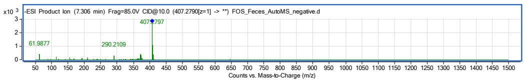	Standard
HCA isomer 1	5.9	407.2814	407.2814		F		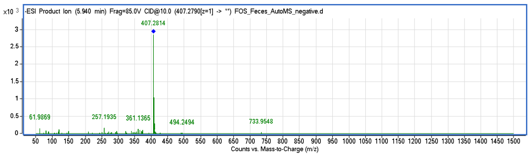	[13]
HCA isomer 2	8.1	407.2793	407.2793		F		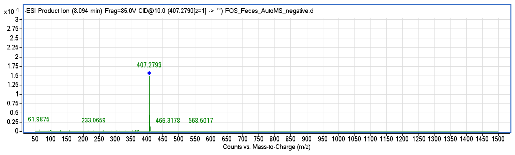	[13]
HCA isomer 3	8.4	407.2806	407.2806		F		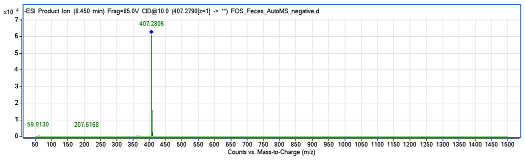	[13]
CA	8.7	407.2809	407.2809	P	F		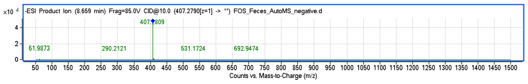	Standard
GLCA	11.1	432.3121	74.0242	P			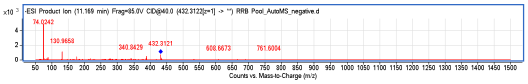	Standard
Glyco-7-oxo-LCA isomer 1	4.6	446.2900	74.0247	P		U	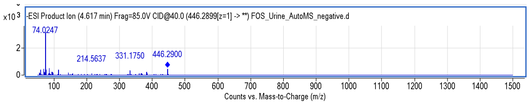	No reference
Glyco-7-oxo-LCA isomer 2	5.6	446.2910	74.0239	P			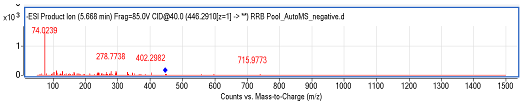	No reference
Glyco-7-oxo-LCA isomer 3	7.9	446.2894	74.0243	P	F	U	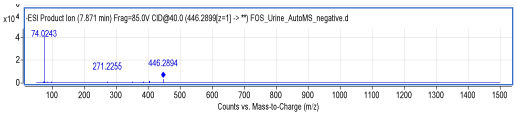	No reference
GUDCA isomer 1	6.7	448.3060	74.0244	P	F		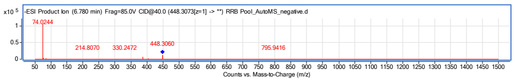	Standard

GUDCA isomer 2	7.6	448.3068	74.0243	P	F	U	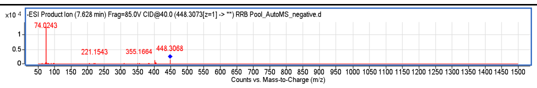	Standard
GCDCA	8.9	448.3059	74.0242	P	F		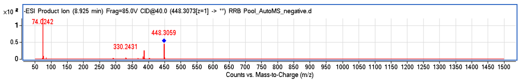	Standard
GDCA	9.3	448.3066	74.0246	P	F		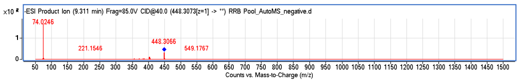	Standard
3-oxo-LCA-sulfate isomer	8.1	453.2292	96.9599		F		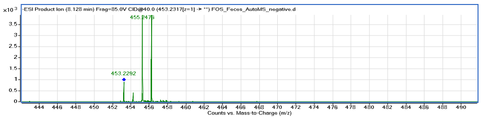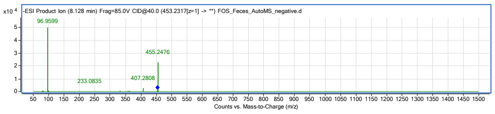	[13]
LCA-sulfate	8.3	455.2478	96.9598		F		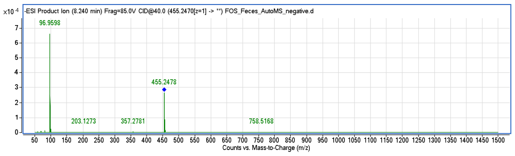	[13]
Glyco-12-oxo-CDCA isomer	3.9	462.2860	74.0239	P			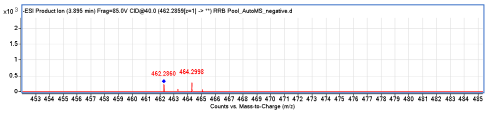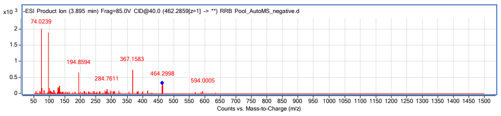	No reference
GHCA isomer 1	3.0	464.3006	74.0240	P		U	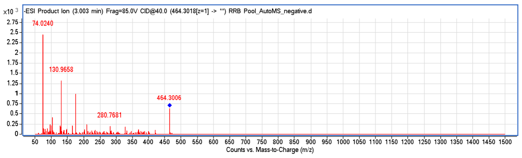	[13]
GHCA isomer 2	3.4	464.3001	74.0245	P		U	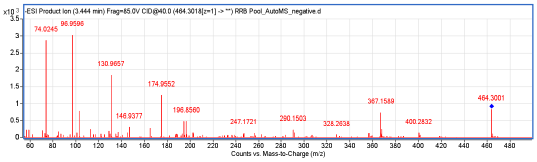	[13]
GHCA	4.7	464.3006	74.0242	P		U	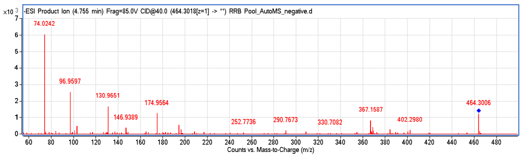	[13]
GCA	6.9	464.3023	74.0244	P	F		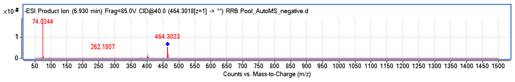	Standard

12-oxo-LCA-sulfate	3.3	469.2265	96.9599		F		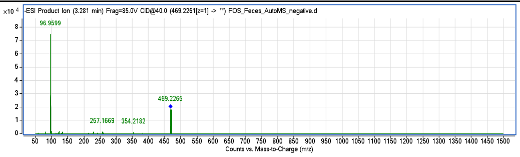	[13]
CDCA-sulfate isomer 1	3.8	471.2426	96.9598		F		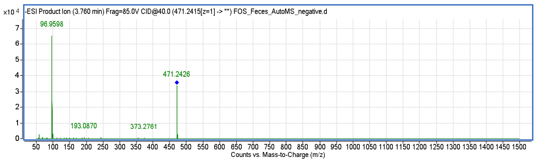	[13]
CDCA-sulfate isomer 2	6.5	471.2433	96.9597		F	U	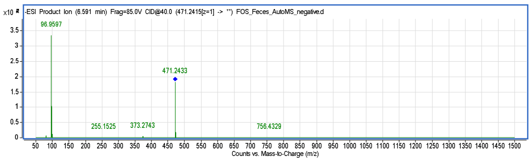	[13]
CDCA-sulfate isomer 3	7.5	471.2425	96.9598	P	F	U	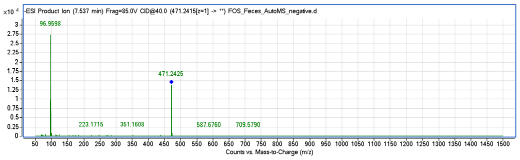	[13]
12-oxo-CDCA-sulfate isomer 1	2.6	485.2220	96.9598		F		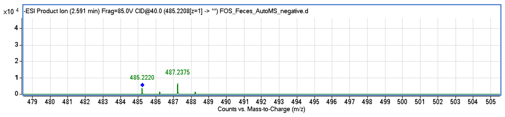 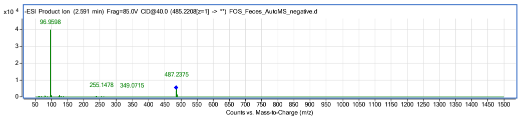	[13]
CA-sulfate isomer 1	2.8	487.2377	96.9597		F		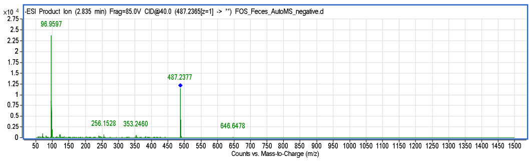	[13]
CA-sulfate isomer 2	3.5	487.2368	96.9595		F		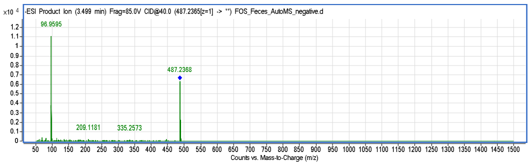	[13]
TCDCA isomer 1	7.0	498.2896	79.9571	p			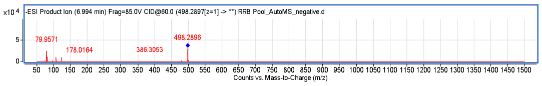	Standard
TCDCA isomer 2	7.7	498.2892	79.9567	P			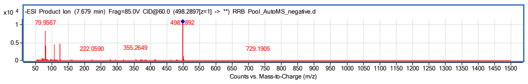	Standard
TCDCA isomer 3	8.2	498.2889	79.9565	P			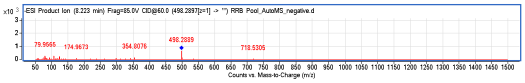	Standard

TCA isomer 1	2.3	514.2837	514.2837/<br>79.9566	P	F		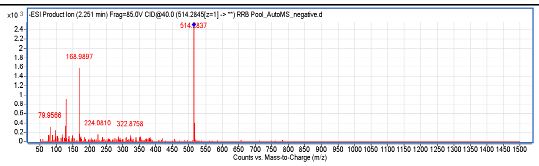	[13,27]
TCA isomer 2	3.2	514.2844	514.2844/<br>79.9573	P			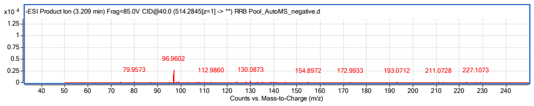<br>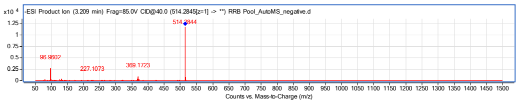	[13,27]
GCDCA-sulfate isomer 1	3.2	528.2627	74.0242/<br>96.9597			U	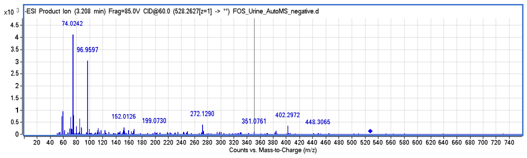	[13]
GCDCA-sulfate isomer 2	7.0	528.2627	74.0245/<br>96.9598			U	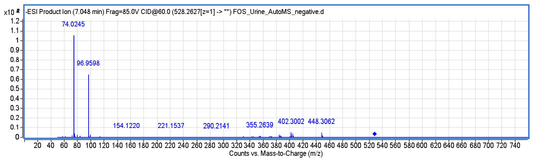	[13]
GCA-sulfate isomer	3.4	544.2591	96.9596/<br>464.3014			U	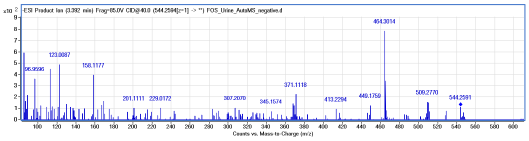	[28]
Glucuronide-12-oxo-LCA isomer 1	5.4	565.3009	389.2712/<br>75.0084			U	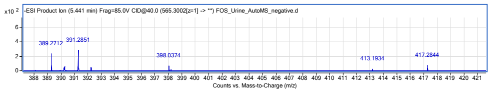<br>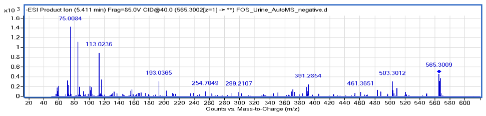	[27]
Glucuronide-12-oxo-LCA isomer 2	5.8	565.2995	389.2685/<br>75.0080	P		U	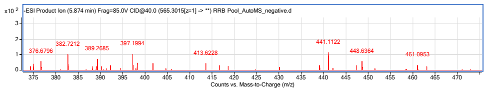<br>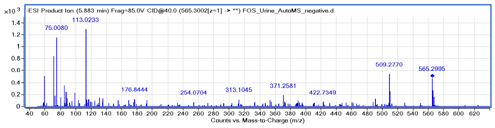	[27]
Glucuronide-12-oxo-LCA isomer 3	6.4	565.3021	389.2704/<br>75.0082	P			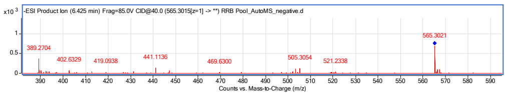<br>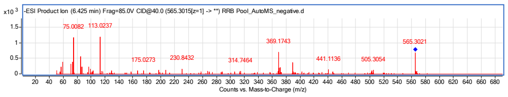	[27]

Glucuronide-CDCA isomer 1	4.2	567.3178	391.2847/ 75.0077		U	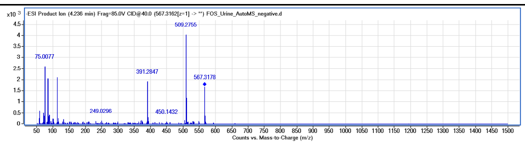	[27]
Glucuronide-CDCA isomer 2	6.7	567.3168	391.2858/ 75.0078	P	U	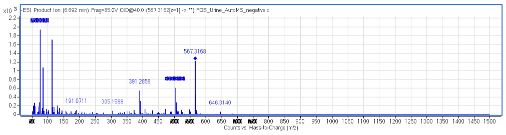	[27,29]
Glucuronide-CDCA isomer 3	8.5	567.3175	391.2845/ 75.0080	P	U	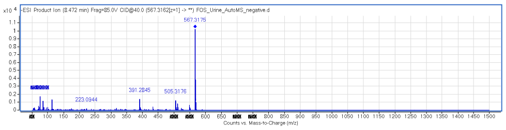	[27,29]
Glucuronide-CA isomer 1	2.6	583.3137	407.2799/ 75.0081	P	U	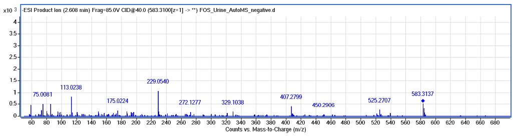	[27,29]
Glucuronide-CA isomer 2	4.0	583.3130	407.2804/ 75.0085		U	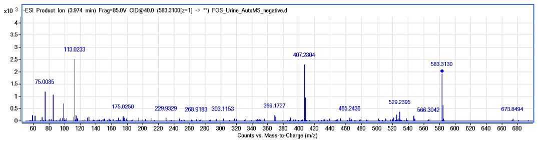	[27,29]
Glucuronide-CA isomer 3	5.3	583.3117	407.2800/ 75.0086		U	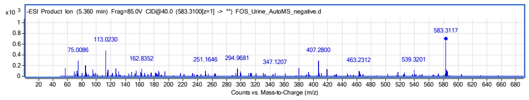	[27,29]
Glucuronide-CA isomer 4	5.9	583.3100	407.2798/ 75.0071		U	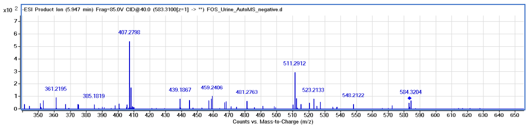 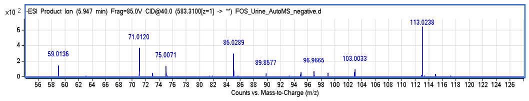	[27,29]
Glucuronide-GLCA	8.6	608.3425	432.3117/74.0242	P		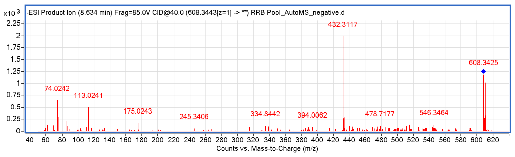	No reference
Glucuronide-GCDCA isomer 1	3.0	624.3400	448.3051/ 74.0245	P	U	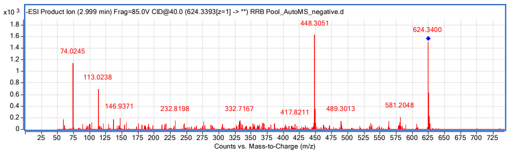	No reference
Glucuronide-GCDCA isomer 2	7.0	624.3388	448.3070/ 74.0246	P	U	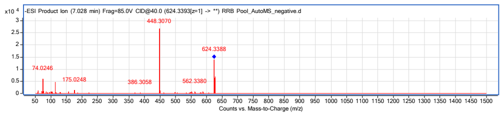	No reference

Glucuronide-GCDCA isomer 3	7.4	624.3385	448.3062/ 74.0245		U	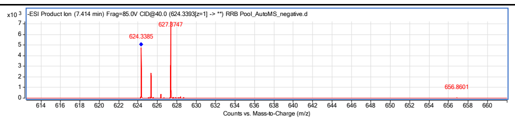 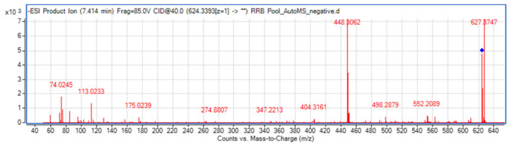	No reference
Glucuronide-GCA isomer 1	2.2	640.3318	464.3014/ 74.0234	P	U	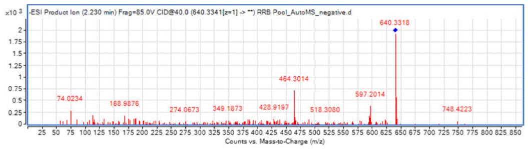	No reference
Glucuronide-GCA isomer 2	2.9	640.3344	464.3017/ 74.0240		U	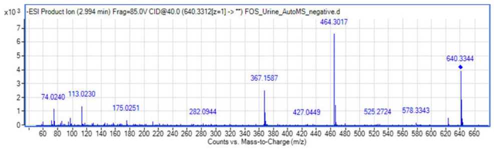	No reference
Glucuronide-GCA isomer 3	3.3	640.3345	464.3019/ 74.0239	P		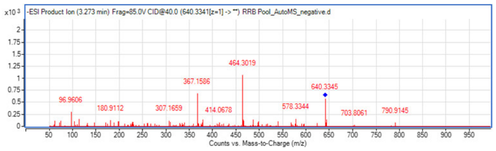 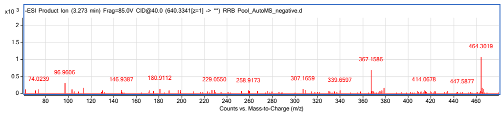	No reference

[1]m/z corresponds to [M-H][1-].

[2]MS/MS fragments: MS/MS fragments listed as Quantifier/Qualifier ion used in the UHPLC-QQQ method. Single transitions means the same ion was used as both quantifier and qualifier ion.

[3]Detected in P, plasma; U, urine; F, feces.

Abbreviations: cholic acid (CA), chenodeoxycholic acid (CDCA), deoxycholic acid (DCA), glycocholic acid (GCA), glycochenodeoxycholic acid (GCDCA), glycodeoxycholic acid (GDCA), glycolithocholic acid (GLCA), glycoursodeoxycholic acid (GUDCA), hyocholic acid (HCA), hyodeoxycholic acid (HDCA), lithocholic acid (LCA), β-muricholic acid (β-MCA), murideoxycholic acid (MuriDCA), taurocholic acid (TCA), taurochenodeoxycholic acid (TCDCA), taurodeoxycholic acid (TDCA), taurolithochoic acid (TLCA), tauroursodeoxycholic acid (TUDCA), taurohyodeoxycholic acid (THDCA), ursodeoxycholic acid (UDCA).

Table S2. BA standards used for quantification of individual BA metabolites.

Standard used for quantification	BA Name
CDCA	CDCA
TCDCA	TCDCA
TUDCA	TUDCA
GUDCA	GUDCA
GDCA	GDCA
THDCA	THDCA
TDCA	TDCA
MuriDCA	MuriDCA
UDCA	UDCA
HDCA	HDCA
CA	CA
	Glucuronide-CA isomer 1
	Glucuronide-CA isomer 2
	CA-sulfate isomer 1
	CA-sulfate isomer 2
GCA	GCA
	Glucuronide-GCA
TCA	TCA
	TCA isomer 1
	TCA isomer 2
	TCA isomer 3
β -MCA	β -MCA
	β -MCA isomer
HCA	HCA
	HCA isomer
GCDCA	GCDCA
	Glucuronide-GCDCA isomer 1
	Glucuronide-GCDCA isomer 2
	GCDCA-sulfate isomer 1
	GCDCA-sulfate isomer 2
	GCDCA-sulfate isomer 3
GLCA	GLCA
	Glucuronide-GLCA
	GLCA-sulfate

TLCA	TLCA
	TLCA-sulfate
LCA	LCA
	LCA-sulfate, isomer 1
	LCA-sulfate, isomer 2
	LCA-sulfate, isomer 3
	LCA-sulfate, isomer 4
	LCA-sulfate, isomer 5
	3-OXO-LCA, isomer 1
	3-OXO-LCA, isomer 2
	3-OXO-LCA, isomer 3
	3-OXO-LCA, isomer 4
	Glyco-3-OXO-LCA, isomer 1
	Glyco-3-OXO-LCA, isomer 2
	Glyco-3-OXO-LCA, isomer 3
	3-OXO-LCA-sulfate, isomer 1
	3-OXO-LCA-sulfate, isomer 2
	3-OXO-LCA-sulfate, isomer 3
	3-OXO-LCA-sulfate, isomer 4
	3-OXO-LCA-sulfate, isomer 5
	3-OXO-LCA-sulfate, isomer 6
	7-OXO-LCA isomer 1
	7-OXO-LCA isomer 2
	7-OXO-LCA isomer 3
	7-OXO-LCA isomer 4
	7-OXO-LCA isomer 5
	7-OXO-LCA isomer 6
	7-OXO-LCA isomer 7
	7-OXO-LCA isomer 8
	Glyco-7-oxo-LCA isomer 1
	Glyco-7-oxo-LCA isomer 2
	Glyco-7-oxo-LCA isomer 3
	Glyco-7-oxo-LCA isomer 4
	Glucuronide-12-oxo-LCA isomer 1
	Glucuronide-12-oxo-LCA isomer 2
	Glucuronide-12-oxo-LCA isomer 3
	3α-hydroxy-6,7-diketo cholianic acid, isomer 1
	3α-hydroxy-6,7-diketo cholianic acid, isomer 2

	3α-hydroxy-6,7-diketo cholianic acid, isomer 3
	9(11),(5β)-cholanic acid-3α-ol-12-one, isomer 1
	9(11),(5β)-cholanic acid-3α-ol-12-one, isomer 2
	9(11),(5β)-cholanic acid-3α-ol-12-one, isomer 3
	9(11),(5β)-cholanic acid-3α-ol-12-one, isomer 4
	9(11),(5β)-cholanic acid-3α-ol-12-one, isomer 5
DCA	DCA
	nor-DCA, isomer 1
	nor-DCA, isomer 2
	nor-DCA, isomer 3
	nor-DCA, isomer 4
	nor-DCA, isomer 5
	7-oxo-DCA isomer 1
	12-oxo-CDCA-sulfate isomer 1
	12-oxo-CDCA-sulfate isomer 2
	Glyco-12-oxo-CDCA isomer 1
	Glyco-12-oxo-CDCA isomer 2
	Glyco-12-oxo-CDCA isomer 3
	Glyco-12-oxo-CDCA isomer 4
	Glucuronide-CDCA isomer 1
	Glucuronide-CDCA isomer 2
	Glucuronide-CDCA isomer 3
	Glucuronide-CDCA isomer 4
	Glucuronide-CDCA isomer 5
	CDCA-sulfate isomer 1
	CDCA-sulfate isomer 2
	CDCA-sulfate isomer 3
	CDCA-sulfate isomer 4

Table S3. Nutritional composition of control and strawberry beverage (per 50 g portion)[1]

	Control	Strawberry
Calories (kcal)	180	172
Total carbohydrate (g)	44	39
Total sugar (g)	27	29

Dietary fiber (g)	5.2	8.0
Anthocyanin (mg)	0	141.7
Ellagitannins (mg)	0	160.2
Phenolic acids (mg)	0	13.6
Flavan-3-ols (mg)	0	99.0
Flavonols (mg)	0	36.2

[1] Data obtained from Huang *et al*., 2020 [50].
